# PlantCTCIP: Chromatin Interaction Prediction Using Convolutional Neural Network and Transformer in Plants

**DOI:** 10.1111/pbi.70586

**Published:** 2026-02-12

**Authors:** Zhenye Wang, Siyu Zhou, Ze Guo, Zilan Ning, Jiaqi Cai, Ran Zhao, Ao Xie, Quan Li, Jiangling Zhang, Yongsheng Zhao, Peizhang Li, Haiping Si, Jianbing Yan, Yong Peng, Jianxiao Liu

**Affiliations:** ^1^ National Key Laboratory of Crop Genetic Improvement Huazhong Agricultural University Wuhan China; ^2^ College of Information and Management Science Henan Agricultural University Zhengzhou China; ^3^ Hubei Key Laboratory of Agricultural Bioinformatics Huazhong Agricultural University Wuhan China; ^4^ College of Informatics Huazhong Agricultural University Wuhan China; ^5^ College of Plant Science & Technology Huazhong Agricultural University Wuhan China; ^6^ Hubei Hongshan Laboratory Wuhan China

**Keywords:** chromatin interactions, deep learning, plants, regulatory elements, TF collaboration

## Abstract

Chromatin interactions establish spatial proximity between distant regulatory elements and their target genes, significantly influencing gene expression, and phenotypic traits. In this study, we present a plant chromatin interaction prediction model called PlantCTCIP based on Convolutional Neural Networks and Transformer. PlantCTCIP demonstrated superior performance compared to the conventional models. Specifically, PlantCTCIP improved the average AUC of chromatin interaction predictions by 14.56% across the four species in PPI (proximal promoter interaction) mode. Similarly, PlantCTCIP improved the average AUC of chromatin interaction predictions by 9.6% in the PDI (distal promoter interaction) mode. We constructed genome‐wide chromatin interaction maps for four plants (maize, rice, cotton and wheat) through PlantCTCIP, further used the Hi‐C experiment to validate correctness of the predicted PPIs and PDIs. Some key motifs that influence chromatin interactions are identified, and they are significantly enriched in expression quantitative trait loci (eQTLs) and open chromatin regions. We also analysed the enrichment and species specificity of the transcription factors (TF) and synergistic network of TFs that affect PPIs and PDIs of four crops. Using cloned genes (*ZmRAVL1*, *ZmRPG*, *ZmRap2.7* and *GaFZ*) of maize and cotton as examples, PlantCTCIP can assist in identifying target genes regulated by distal elements and mining functional sites combined with chromatin conformation capture (3C) experiments. This research helps to analyse the regulatory mechanism of gene expression and provides novel perspectives for intelligent design breeding of diverse crops. PlantCTCIP is available at http://www.plantctcip.com.

## 1 Introduction

Distal elements interact with target genes through chromatin interactions, thereby affecting gene expression. Researches on three‐dimensional chromatin interactions have made significant progress in animals and plants (Ouyang et al. 2023). A deep understanding of the potential mechanisms related to chromatin such as chromatin interactions, chromatin loops and topologically associated domains (TADs) is crucial for comprehensively understanding gene transcriptional regulation (Whalen et al. 2016). In recent years, the three‐dimensional genomics technologies (Hi‐C Micro‐C and ChIA‐PET) have been gradually applied in plants. However, the chromatin interaction related datasets of maize (Dong et al. 2020), rice (Liu et al. 2017), cotton (Wang et al. 2018) and wheat (Ramírez‐González et al. 2018) are limited. It restricts further analysis of chromatin interaction patterns and the identification of sequence features that affect chromatin interactions. Therefore, analysing the interaction mechanism of plant 3D genomes through machine learning has important research significance.

By integrating genomic sequence features with epigenetic data, researchers have developed a series of machine learning models to predict human enhancer‐promoter interactions and TAD boundaries. The typical models include SPEID (Singh et al. 2019), ChINN (Cao et al. 2021), DeepTACT (Li et al. 2019) and SEPT (Jing et al. 2020). These methods primarily employ the classical deep learning approaches (such as CNN and Recurrent Neural Network [RNN]) to predict enhancer‐promoter interactions. Although RNN and LSTM are commonly used for genome sequence modelling, their inherent sequential processing mechanisms make it difficult to alleviate gradient vanishing problems when dealing with long sequences. The recursive structure of RNN and LSTM is difficult to effectively capture the dependencies between distant features. At present, there are few reports on the prediction of chromatin interactions related to plants such as wheat, maize, rice and cotton. This limitation relates to the factors of the lack of high‐quality 3D genomic datasets and complex genome structure (such as high proportions of repetitive sequences and frequent polyploidization events) of plants. For instance, the complex sequence architectures of hexaploid wheat and tetraploid cotton elevate the challenges of constructing high‐precision chromatin interaction models and identifying sequence features. Most existing chromatin interaction prediction models are only trained and tested in a single species, leading to low accuracy when applied to other species. The limited generalisation ability of predictive models also restricts the accuracy of analysing sequence features that affect chromatin interactions. Leveraging reference genome sequences, chromatin interactions and multiple omics data of four plants (maize, rice, wheat and cotton), this study conducted researches in the following aspects.

(1) We developed a plant chromatin interaction prediction model of PlantCTCIP (Plant Chromatin Interaction Prediction using CNN‐Transformer). This model utilises the self‐attention mechanism in the transformer to capture the relationships between each feature and the features of other positions in the sequence. The fusion of CNN and transformer helps to capture multi‐scale features of the local and global dimensions in DNA sequences. The AUC and PR values of PlantCTCIP are significantly higher than those of the existing methods in both PPIs and PDIs modes. We constructed genome‐wide chromatin interaction maps of maize, rice, wheat and cotton using PlantCTCIP, and further used Hi‐C experiment to validate the correctness of the predicted PPIs and PDIs in four sub‐regions.

(2) We used PlantCTCIP to identify important motifs that affect PPIs and PDIs of four crops. The identified motifs exhibit significant species specificity and are significantly enriched in expression quantitative trait loci (eQTLs) and open chromatin regions. We also analysed the enrichment and species specificity of the transcription factors (TF) and the synergistic network of TFs that affect PPIs and PDIs of four crops.

(3) Taking the reported maize genes of *ZmRAVL1*, *ZmRPG*, *ZmRap2.7* and cotton gene of *GaFZ* as examples, we integrated 3C experiments to demonstrate that PlantCTCIP can identify target genes that interact with specific regulatory regions and mine functional sites in distal regulatory elements. We have also developed an online service platform of multi‐plant chromatin interaction prediction (http://www.plantctcip.com/).

## 2 Results

**FIGURE 1:**
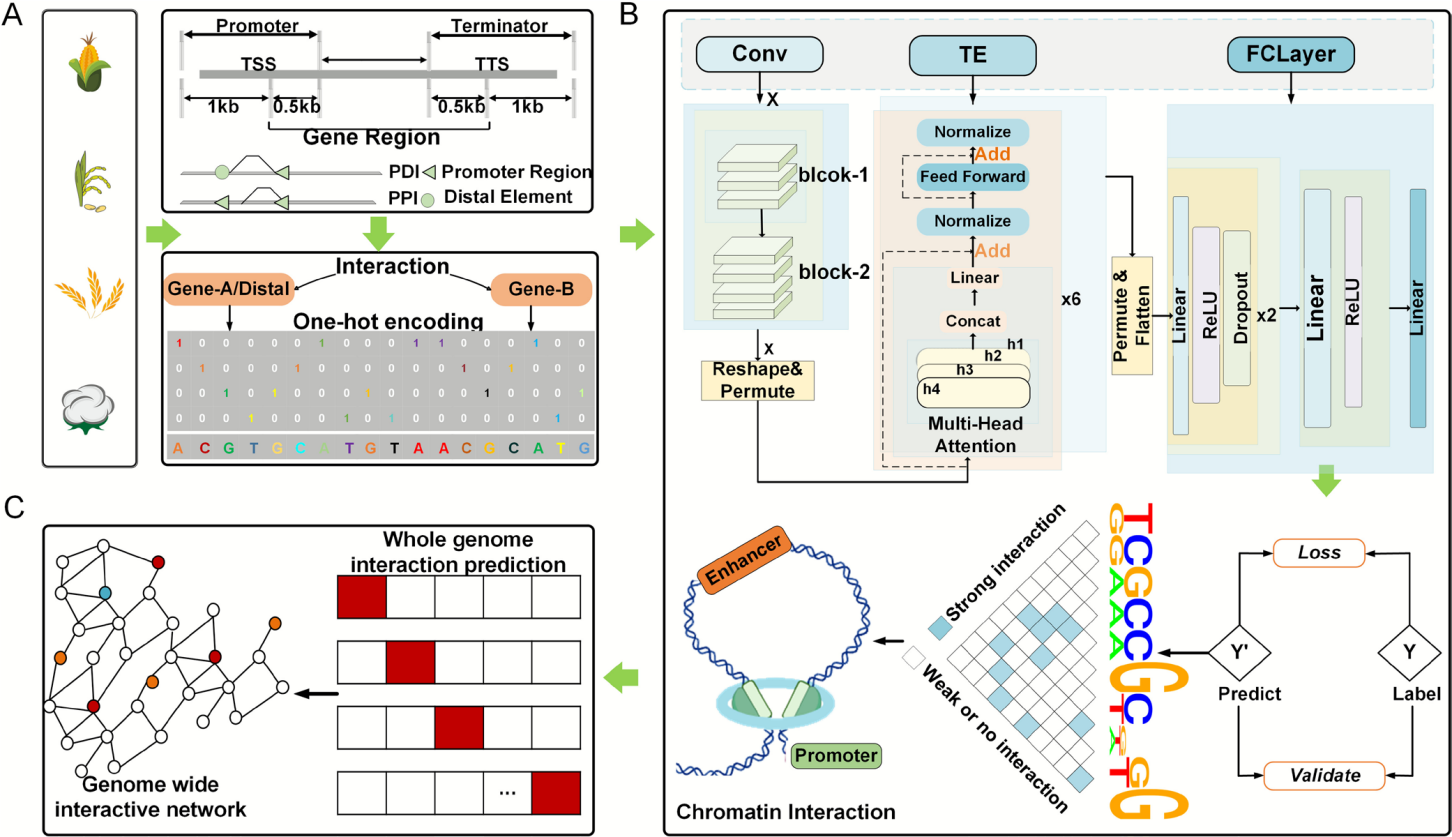
The workflow of PlantCTCIP. (A) Two kinds of chromatin interactions of four crops: PPIs and PDIs. The sequence of a gene involves 1 kb upstream and 0.5 kb downstream of TSS, and 0.5 kb upstream and 1 kb downstream of TTS. (B) Three modules in PlantCTCIP: feature extraction using CNN, temporal and distal feature extraction using Transformer, and chromatin interactions prediction. (C) Use PlantCTCIP to predict the whole genome chromatin interactions of four crops.

This study collected chromatin interaction data of four plants: maize (Peng et al. 2019; Li et al. 2019; Sun et al. 2020), cotton (Huang et al. 2022), wheat (Yuan et al. 2022) and rice (Zhao et al. 2019; Zhang et al. 2016). Two types of chromatin interactions were considered in these four species: (i) Promoter proximal region interaction (PPIs) and (ii) Promoter‐distal element interactions (PDIs) (Washburn et al. 2019; Wang et al. 2024). We determine the sequence length based on the prior reported information and data features. The most existing studies of genome sequences analysis based on deep learning use the gene promoter length of 1.5 kb to do experiments (Washburn et al. 2019; Wang et al. 2024). In addition, the length of sequences involved in chromatin interactions is mainly in the range of 1–2 kb (Peng et al. 2019). We tested the prediction effects of three sequence lengths (1, 1.5 and 2 kb) of PlantCTCIP. In our experiments, the length of the distal element sequence is 1.5 kb, and the length of gene sequence is 3 kb (including 1.5 kb upstream and downstream of TSS and TTS) (Figure S1A). The length of the distal element sequence in control group 1 is 1 kb, and the length of the gene sequence is 2 kb (including 1 kb upstream and downstream of TSS and TTS) (Figure S1B). The length of the distal element sequence in control group 2 is 2 kb, and the length of the gene sequence is 4 kb (including 2 kb upstream and downstream of TSS and TTS) (Figure S1C). The experimental results show that PlantCTCIP has better prediction accuracy than control group 1 and control group 2 when the length of the distal element sequence is 1.5 kb and the length of the gene sequence is 3 kb (Figure S2). Therefore, we set the length of distal element and target gene sequences to 1.5 and 3 kb to conduct experiments of four species. The DNA sequence for a specific gene involves 1 kb upstream and 0.5 kb downstream of the transcription start site (TSS) and 0.5 kb upstream and 1 kb downstream of the transcription termination site (TTS) (Figure 1A). The sequence length of intergenic regions used in this study mainly ranged from 1 to 2 kb and a 1.5‐kb sequence was selected as the distal regulatory element sequence using truncation and padding methods (Section 4). The leaf data of maize B73 at the seedling stage includes 43 865 pairs of PPIs and 14 703 pairs of PDIs. The leaf data of wheat China Spring at the seedling stage includes 126 870 pairs of PPIs and 152 358 pairs of PDIs. The leaf data of cotton A2 at the seedling stage includes 42 646 pairs of PPIs and 55 470 pairs of PDIs. The rice varieties of MH63 and ZS97 involve 39 147 and 40 423 pairs of PPIs, respectively (Table S1).

We developed PlantCTCIP, a plant chromatin interaction prediction model based on Convolutional Neural Networks and Transformers (Figure 1B). In this framework, the convolutional neural network (CNN) module extracts sequence features from the encoded chromatin sequences while performing dimensionality reduction. The Transformer module utilises long‐range contextual information to capture dependent relationships among sequence features. The output layer employs two activation functions: a linear function and a sigmoid function. The linear function generates binary predictions (0 or 1) that are used to indicate interaction status, while the sigmoid function outputs interaction probabilities (0–1). The predictions exceeding the probability threshold (0.5) are classified as interactions (category 1). We used PlantCTCIP to predict genome‐wide chromatin interactions for four plants through two complementary approaches (Section 4).

### 2.1 PlantCTCIP Has Better Accuracy and Generalisation Ability in Predicting Chromatin Interactions

**FIGURE 2:**
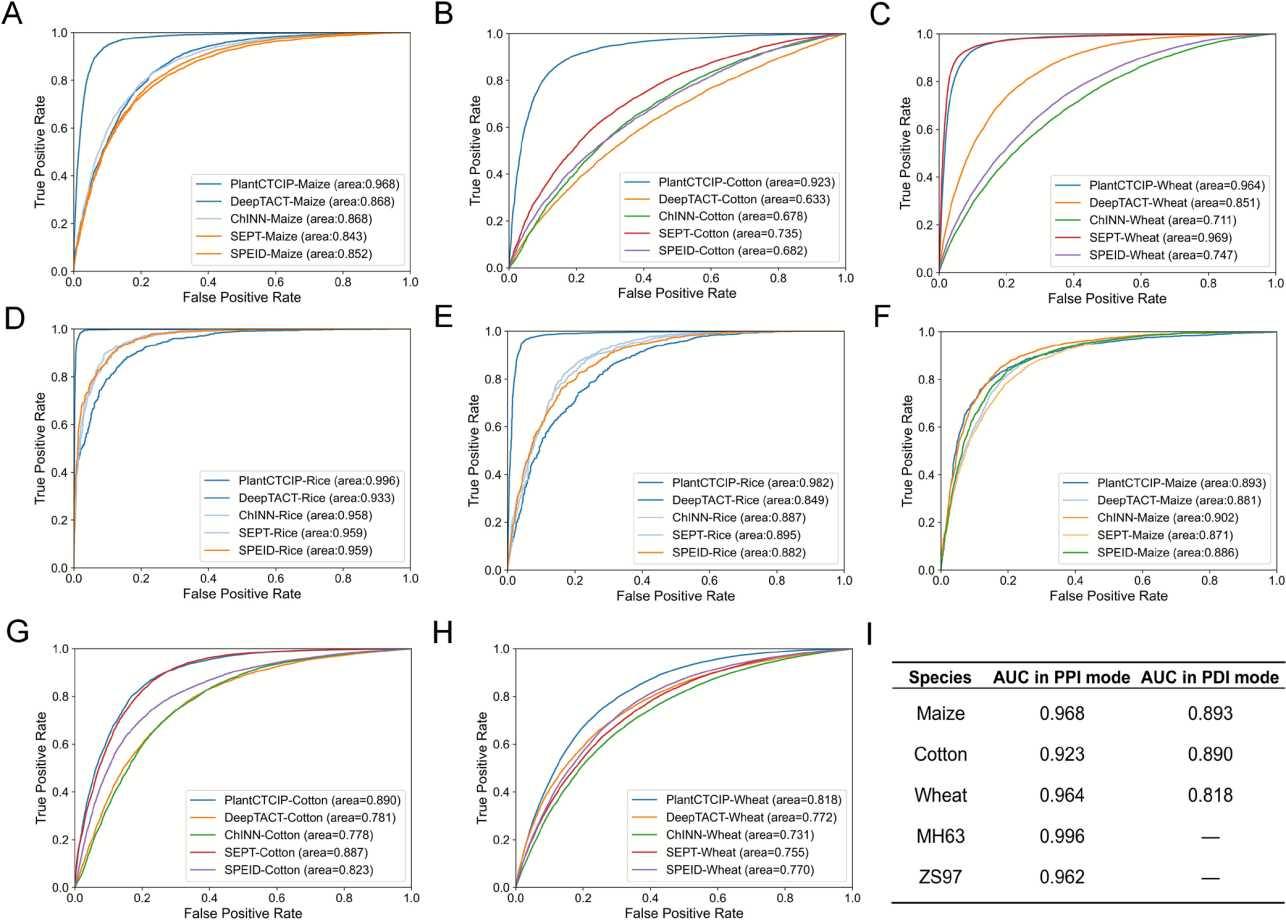
Performance of PlantCTCIP in predicting multi‐plant chromatin interactions of Shoot dataset. (A–E) The AUC value comparison results of PlantCTCIP, DeepTACT, ChINN, SEPT, SPEID in maize, cotton and wheat in PPIs mode. (F–H) The AUC value comparison results of PlantCTCIP, DeepTACT, ChINN, SEPT, SPEID in maize, cotton and wheat in PDIs mode. (I) The summary of AUC values in predicting chromatin interactions of different species in PPIs and PDIs modes of PlantCTCIP.

In order to evaluate the performance of PlantCTCIP in predicting chromatin interactions, this study conducted experimental comparisons of four commonly used models for predicting chromatin interactions in PPIs and PDIs modes. The four comparison models are DeepTACT (Li et al. 2019), ChINN (Cao et al. 2021), SEPT (Jing et al. 2020) and SPEID (Singh et al. 2019) (Section 4). Prediction results showed that PlantCTCIP achieved AUC values of 0.968 for maize, 0.923 for cotton, 0.964 for wheat and 0.996 for rice in PPIs mode. They were significantly higher than the existing four models of four plants. The average AUC values increased by 10.1%, 24.6%, 14.45% and 9.1%, respectively (Figure 2A–E). Similarly, PlantCTCIP has an increase in AUC values of 2.5%, 14.4% and 12% in maize, cotton and wheat compared to the other four models in PDIs mode (Figure 2F–H). As depicted in Figure 2I and Figures S3–S5, PlantCTCIP exhibits higher prediction accuracy in the PPIs mode than the PDIs mode across different crops. This is mainly related to the accuracy of the datasets of the two modes. These results demonstrate that PlantCTCIP has high prediction accuracy and universality among different species. In order to evaluate the effectiveness of PlantCTCIP in predicting chromatin interactions among different ploidy species, we used the PlantCTCIP to predict chromatin interactions in the sub‐genome of polyploid species (taking wheat as an example). The results showed that the prediction accuracy of PlantCTCIP in the three sub‐genomes of wheat was similar (Figure S6). This indicates that the sequence similarity between homologous chromosomes results in similar prediction accuracy and can equally effectively capture the characteristics of different sub‐genomes. To evaluate the ability of PlantCTCIP to predict chromatin interaction across tissues, we used five maize datasets with different developmental stages and tissues (Peng et al. 2019; Li et al. 2019; Sun et al. 2020) to predict chromatin interaction across tissues and stages. The results show that the prediction accuracy of chromatin interaction between different tissues is 0.63–0.90, and it means that PlantCTCIP has better chromatin interaction prediction ability across tissues and stages (Figure S7).

To verify the generalisation ability of PlantCTCIP in predicting chromatin interactions across species, we utilised transfer learning to conduct cross species chromatin interaction prediction. By using the ‘pre‐training and fine‐tuning’ paradigm, the high‐dimensional feature representations learned by PlantCTCIP in the source species are transferred and applied to the target species. The results indicate that transfer learning can significantly improve the prediction accuracy of PlantCTCIP compared to directly predicting chromatin interactions. To evaluate the computational efficiency advantages of transfer learning, we compared the training and computational time consumption of using transfer learning to predict chromatin interactions across species. The experimental results show that transfer learning strategies significantly accelerate the convergence process. It leads to a notable reduction in the training and computation time of PlantCTCIP (Figure S8).

### 2.2 PlantCTCIP Achieves High‐Precision Prediction of the Whole Genome PPIs in Four Plants

**FIGURE 3:**
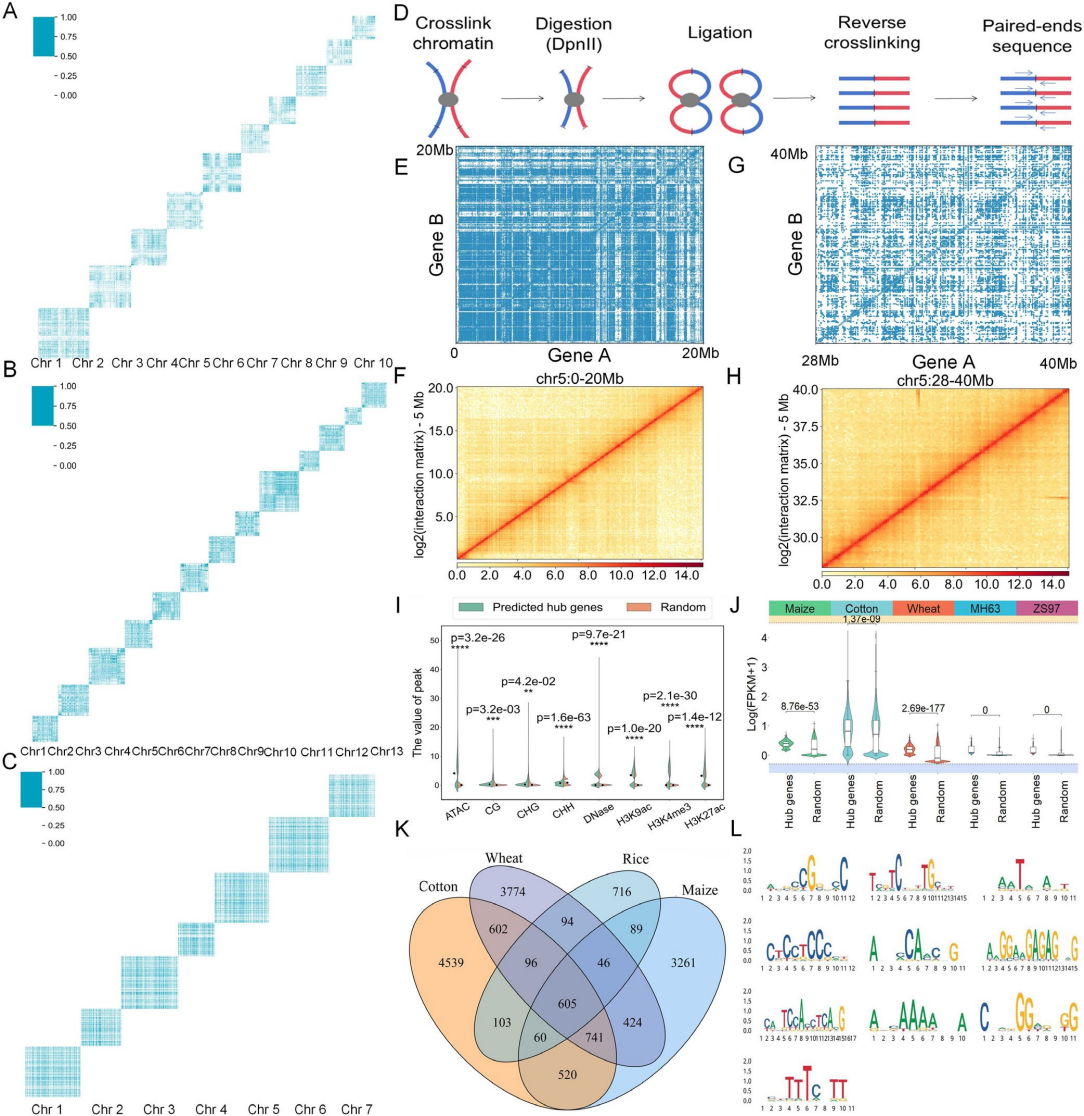
The predicted chromatin interactions in whole genome and the identified high interaction genes and important motifs in PPIs mode using PlantCTCIP. (A–C) The predicted chromatin interactions across the whole genome of maize (A), cotton (B) and wheat (C) using PlantCTCIP. (D) The workflow of Hi‐C experiment. (E) The predicted chromatin interaction map of chr5: 0–20 Mb in maize using PlantCTCIP. (F) The maize chromatin interactions of chr5: 0–20 Mb obtained using Hi‐C experiment. (G) The predicted chromatin interaction map of chr5: 28–40 Mb in maize using PlantCTCIP. (H) The maize chromatin interactions of chr5: 28–40 Mb obtained using Hi‐C experiment. (I) The enrichment comparison results of the selected high interaction genes and control genes in eight omics data (ATAC, CG, CHG, CHH, DNase, H3K27ac, H3K4me3 and H3K9ac) in the PPIs mode of maize. (J) The expression comparison results of high interaction genes and control genes of four plants in PPIs mode. (K) The number of important motifs that affect chromatin interaction of four crops in PPIs mode, as well as 605 conserved motifs in the four crops. (L) Core seq logo of 10 categories obtained by clustering 605 conserved motifs.

We used PlantCTCIP to predict chromatin interactions in the whole genome of four plants (maize, cotton, wheat and rice), thus to supplement the results of biological experiments. Peng et al. (2019) reported that 98% of chromatin interactions in the whole maize genome are intra chromosomal interactions. In addition, the physical distance between distal elements and genes of different species in the experimental data of constructing the chromatin interaction prediction model is much < 2 Mb (Figure S12). Specifically, we input any two gene sequences in a specific chromosome into the PlantCTCIP model, and then obtain a chromatin interaction panorama in each chromosome of different crops (Figure 3A–C). In order to further verify the reliability of the chromatin map predicted by PlantCTCIP, we used maize B73 seedling leaves to do Hi‐C sequencing (Figure 3D). Taking maize chromosome 5 for example, we used PlantCTCIP to do prediction and obtained chromatin interaction maps of two regions (chr5: 0–20 Mb and chr5: 28–40 Mb) (Figure 3E,G), and the maps display distinct TADs (Topologically Associated Domains) features. Furthermore, we compared the prediction results of PlantCTCIP in the two target regions with the Hi‐C experimental results. The results showed that the interaction hotspots identified by PlantCTCIP exhibited a consistent interaction pattern in the Hi‐C experimental results (Figure 3F,H), validating the prediction reliability of PlantCTCIP. In order to explore the molecular characteristics of high interaction genes in the predicted results, the number of genes that interact with the target gene is denoted as the degree of the target gene. We selected the top 1% and top 1500 genes and OCRs with high chromatin interaction for sequence feature and motif analysis. The results indicate that selecting the top 1500 genes and OCRs can balance the coverage of chromatin interaction samples with the interpretability analysis of the model. We analysed the molecular characteristics of the top 1500 high interaction genes (hub genes) of different crops. Compared with non‐interacting or low interacting genes (randomly repeated 50 times), high interacting genes have higher expression values, higher GC content, longer gene length, more exons, higher openness in the region where the gene is located, and enriched with more active histone modification signals (Figure 3I,J; Figure S9).

We further utilised two methods of DeepLIFT and integrated gradients to mine important motifs that affect chromatin interaction of four crops in PPIs mode. In maize, cotton, rice and wheat, it detects 5746, 7266, 1809 and 6382 important motifs affecting chromatin proximal interactions, respectively (the threshold of the continuous gradient was set to 0.5) (Figure 3J). Most of the detected motifs are specific motifs, with 605 motifs being conserved across different crops. We further clustered 605 conservative motifs to obtain 10 important core motifs (Figure 3K). We employed the TOMTOM platform (Bailey et al. 2015) to compare the identified motifs in PPIs mode with the database of PlantTFDB. It can be seen that the important motifs identified by PlantCTCIP are highly consistent with the previously reported motifs (Figure 3L; Figure S10 and Table S3). Specifically, 7 important motifs are found to be involved in regulating gene chromatin interaction, expression and plant growth and development, etc. For instance, the motif CGTGCCGTSC is reported to be located on the Emb5 promoter related to maize seed development, and it enhances the expression of the promoter (Yu et al. 2010). In addition, the motif GCWGCWGC is a cis‐regulatory element involved in iron excess response in rice (Kakei et al. 2021). A more important motif is SCGCGCGS, and it interacts with transcription factor E2F, participates in regulating gene expression and involving in iron overload response (Kakei et al. 2021) (Table 1). Therefore, the motifs identified by PlantCTCIP have important biological significance.

**TABLE 1:** The concrete information of the identified motifs of the top 1500 highly interactive genes in PPIs mode.

Motifs	Seq‐logo	Functions	References
CGTGCCGTSC	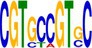	Located on the Emb5 promoter related to maize seed development, it enhances the activity of the promoter.	Yu et al. (2010)
TATATATA	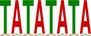	TATA box, RNA polymerase binding site, determines the initiation of transcription.	Bernard et al. (2010)
AACCAAAC	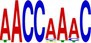	The binding site of Arabidopsis AtMYB4, located near the MYB4 promoter, it is involved in phenylpropane metabolism and stress resistance.	Mitra et al. (2019)
GCWGCWGC	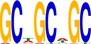	A *cis*‐regulatory element involved in iron excess response in rice.	Kakei et al. (2021)
CAACAACA	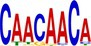	Associated with multiple transcription factors, participating in plant growth and development, stress response and hormone signalling.	Mohanty (2021)
SCGCGCGS	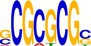	Interactions with E2F transcription factor, involved in regulating gene expression and iron overload response.	Kakei et al. (2021)
AACAACA	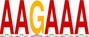	It exists in the transcription terminator of maize gene ZmUbi1, located 14 nucleotides upstream of poly (A) in the 3′ end processing of mRNA, helping to determine the position of the polyadenylation site.	Wang et al. (2020)

To analyse the characteristics and biological functions of the motifs identified by PlantCTCIP in PPIs mode. Firstly, we located the physical location of each motif in the chromosome and matched motifs with the published eQTLs (Liu et al. 2020). The following two processing modes are used as controls: (1) Removing motif sequences from PPIs sequences and selecting sequences with equal length from the remaining PPIs sequences. (2) Removing PPIs sequences from the whole reference genome and selecting sequences from the remaining genome sequences. Compared with the control, the motifs identified by PlantCTCIP are significantly enriched with eQTLs (Figure S11A,B) (****p < 0.0001, *t*‐test). We conducted 100 repeated experiments in the above two controls to reduce the error introduced by randomised experiments. Moreover, we matched these motifs with the open chromatin regions reported in 26 lines of the NAM population (Woodhouse et al. 2021). The results show that the motifs identified by PlantCTCIP are significantly enriched in the open chromatin regions identified in the NAM population (Figure S11C,D) (****p < 0.0001, *t*‐test). The above results indicate that the identified motifs that affect chromatin interaction play a crucial role in regulating gene expression.

In this study, we have mined the conserved motifs (totaling 605 motifs in PPI mode and 170 motifs in PDI mode) by PlantCTCIP in maize, cotton, rice and wheat. We divided all the motifs mined by PlantCTCIP into different enrichment patterns, as shown in the following: The motifs detected by PlantCTCIP are positionally anchored in the 6 kb (3 kb per gene) sequence of PPI sequence, and the distribution pattern of motifs can be divided into 5 categories: (1) slightly enriched near 250 bp downstream of TSS; (2) highly enriched near 250 bp downstream of TSS; (3) slightly enriched in TSS and highly enriched in TTS; (4) only significantly enriched at specific sites (TSS and TTS); (5) distributed evenly throughout the entire sequence. The majority of motifs are enriched approximately 250 bp downstream of gene TSS, which is consistent with previous reports (Washburn et al. 2019).

### 2.3 PlantCTCIP Can Reveal the Correlations Between Transcription Factors and Chromatin Proximal Interactions in Plants

**FIGURE 4:**
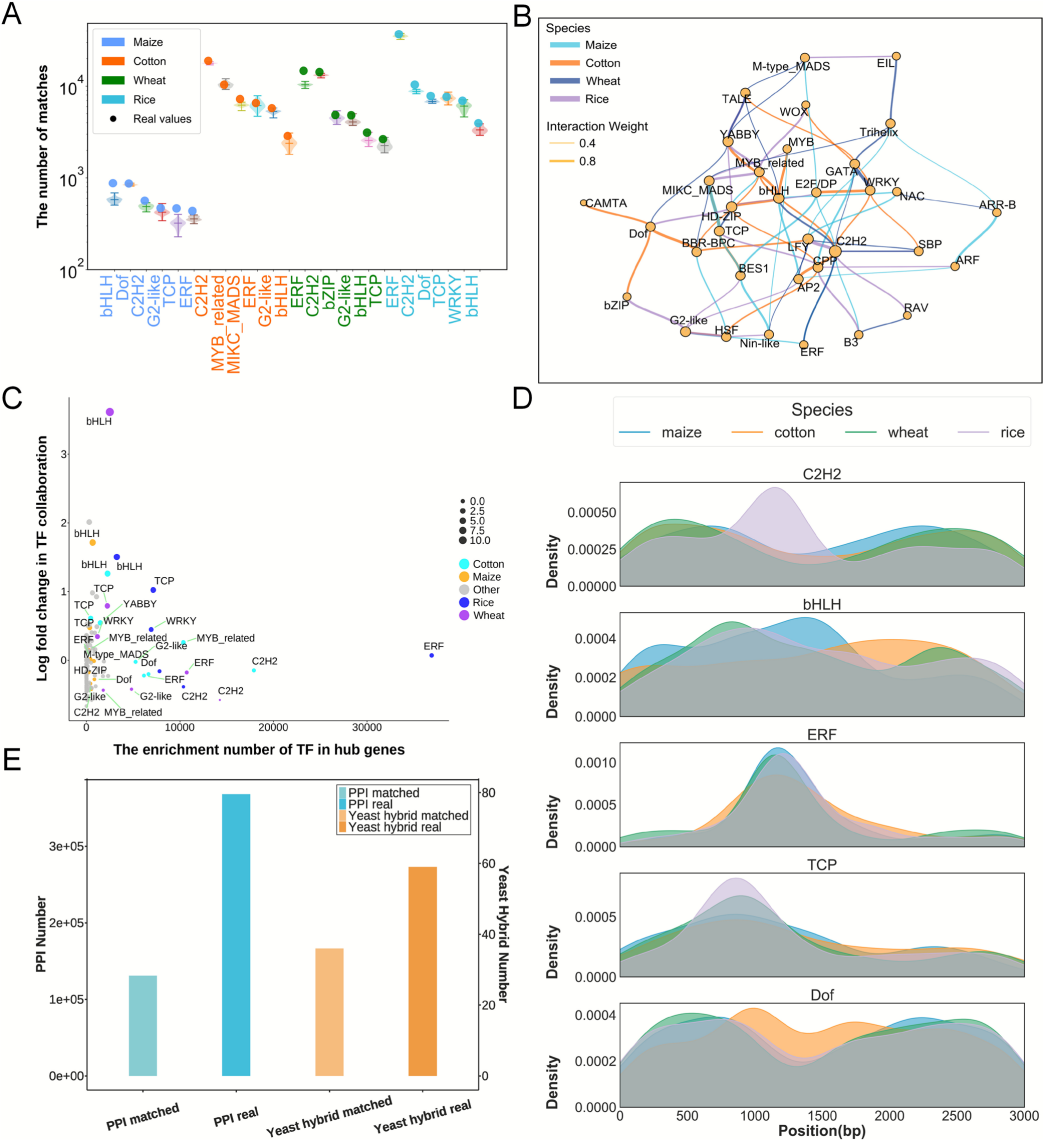
TF enrichment analysis results in PPI sequences predicted by PlantCTCIP in multiple plants. (A) The enrichment comparison results of different types of TFs in hub gene sequence and control group. Comparison method: We randomly selected 1500 genes and extracted their genome sequences, and matched them with the motif sequences of TFs. Repeated the above process 50 times. (B) Examples of TF collaborations in high interaction gene pairs of maize, rice, wheat and cotton in PPIs mode. (C) The correlation between the enrichment number of TFs in hub genes and TF‐TF collaboration of different crops. (D) Density distribution of the transcription factors of C2H2, bHLH, ERF, TCP and Dof in Hub gene sequences of four crops in PPIs mode. (E) The matching number between the chromatin interactions predicted by PlantCTCIP and the interactions using maize bimolecular fluorescence complementation experiments and protein interactions reported in Han et al. (2023).

Gene expression and sequence regulation is a highly complex process involving dynamic changes of transcription factors (TFs) and chromatin structure in plants. TFs bind to gene regulatory elements such as promoters and enhancers through specific DNA binding domains, thereby precisely regulating the expression of downstream genes. Based on the genome‐wide chromatin interactions predicted by PlantCTCIP in the PPIs mode, we obtained the related chromatin interaction sequences of the top 1500 highly interactive genes (hub genes). Based on the previously reported transcription factors (TFs) of four crops in the database of PlantTFDB (Tian et al. 2020), we extracted their corresponding motif sequences and matched them with the sequences of hub genes. Compared with the control, the hub genes with high interactions in the predicted results of PlantCTCIP can be significantly enriched with more TFs (Figure 4A).

We further analysed the distribution of TFs corresponding to two genes in the high interaction gene pairs in different crops. The core TFs mainly include bHLH, Dof, C2H2, TCP, MYB, etc. The results indicate that most TFs associated with hub genes are conserved in maize, rice, cotton and wheat, while some are species‐specific. Notably, the associations between transcription factor CAMTA and Dof only exist in the chromatin interaction sequences of high interactive genes in maize. The associations between the transcription factors ARR‐B and ARF only exist in the chromatin interaction sequences of high‐interaction genes in cotton (Figure 4B). Furthermore, we performed a correlation analysis between the cooperative relationships among transcription factors (TFs) identified in the four crop and the enrichment of these TFs in hub gene sequences. The results show that bHLH family is not only involved in chromatin interactions of all four crops, but is also significantly enriched in hub genes. Additionally, the transcription factor C2H2 is strongly enriched in the hub gene sequences identified in four crops (Figure 4C). We further analysed the distribution patterns of the transcription factors of C2H2, bHLH, ERF, TCP and Dof of the hub gene sequences in the four plants. The results show that ERF and TCP have a consistent distribution pattern in the hub gene sequences of four crops in the PPIs mode. Notably, C2H2 is significantly enriched approximately 250 bp downstream of the transcription start site (TSS) in rice. Similarly, bHLH shows enrichment approximately 250 bp downstream of the TSS in maize, consistent with the results reported by Schlegel et al. (Schlegel et al. 2024) (Figure 4D).

To further investigate the accuracy of the predicted chromatin interactions, we matched maize chromatin interactions predicted by PlantCTCIP with bimolecular fluorescence complementation experiments (BiFC) and protein interaction networks (Han et al. 2023) (Table S2). We collected 59 pairs of publicly reported BiFC data in maize (Han et al. 2023) (Supporting Information), and used PlantCTCIP to predict whether the corresponding gene sequences are interacted or not. The results showed that there are 36 pairs of gene sequences with interaction in PlantCTCIP predicted results, accounting for 61.02%. In addition, we compared the predicted chromatin interactions using PlantCTCIP in maize PPIs mode with the 330 000 pairs of maize protein interactions reported in Han et al. (2023). The results showed that there are approximately 130 000 pairs of chromatin interactions in the two datasets, accounting for 39.4% of the reported 330 000 pairs of chromatin interactions (Figure 4E). These results suggest that chromatin spatial conformation provides strong evidence for protein interactions. In addition, we analysed the enrichment of high‐interaction genes identified by PlantCTCIP in KEGG pathways. The results indicate that the proportion of high interaction genes involved in metabolic pathways is higher than the randomly genes. Notably, *Zm00001d013689* and *Zm00001d017766*, which encode key rate‐limiting enzymes in abscisic acid biosynthesis, having interactions through the prediction of PlantCTCIP. This suggests that these genes may coordinately regulate lutein aldehyde synthesis through chromatin three‐dimensional structural interactions. Additionally, *Zm00001d018181* and *Zm00001d018184* influence the production of flavonoid compound through chromatin proximity‐mediated interactions (Tables S4 and S5).

### 2.4 Analysis of the Whole Genome PDI Prediction Results in Multiple Plants

**FIGURE 5:**
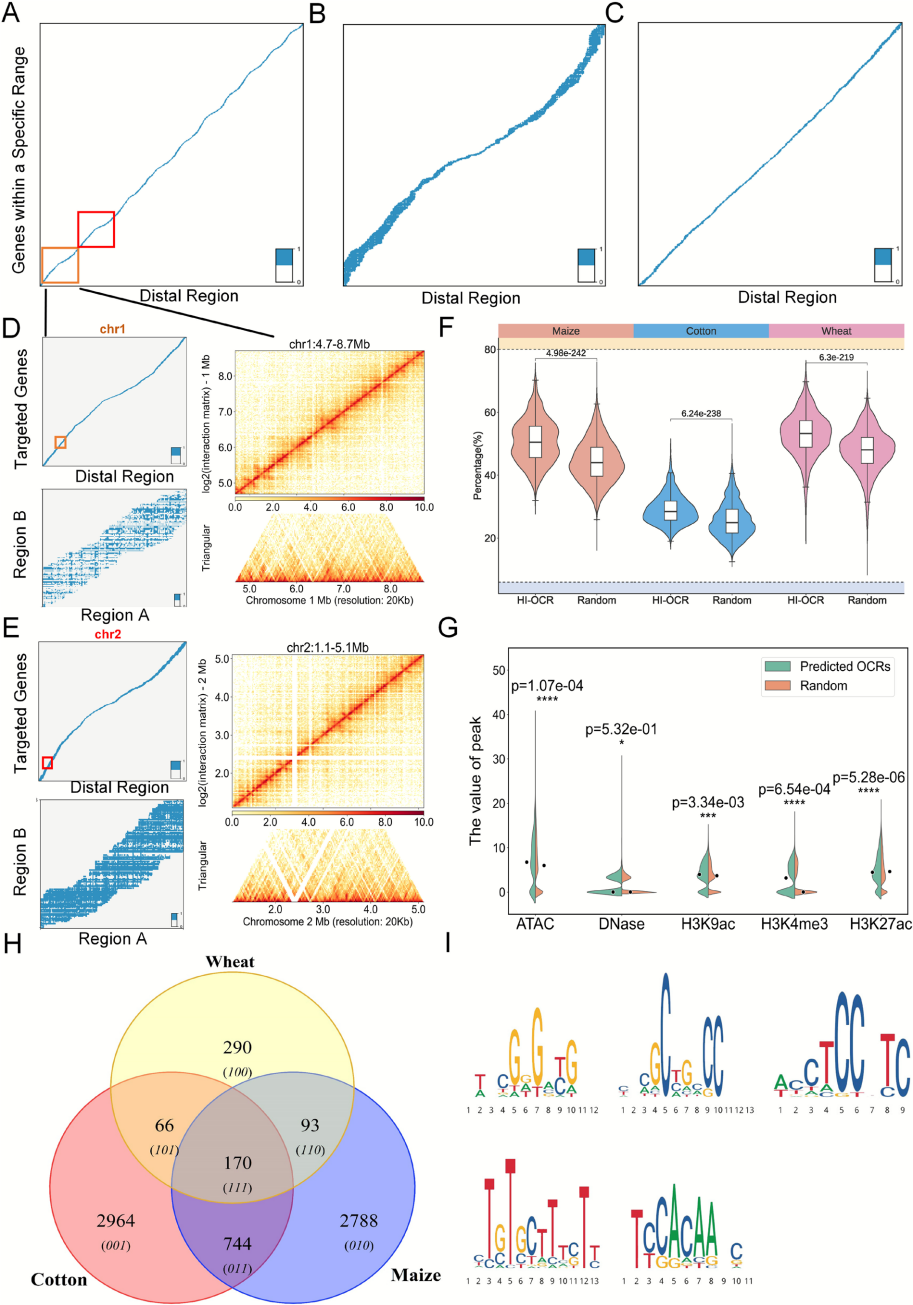
The whole genome chromatin interaction prediction and important motif analysis results of PlantCTCIP in PDI mode. (A–C) The distribution of predicted PDI chromatin interaction of maize (A), cotton (B) and wheat (C) in the whole genome by PlantCTCIP. (D, E) Using the data of maize seedling stage to conduct Hi‐C experiments and verify the chromatin interaction in two regions (chr1: 4.7–8.7 Mb and chr2: 1.1–5.1 Mb). The results indicate that the interactions of Hi‐C experiment are consistent with the predicted results of PlantCTCIP. (F, G) Comparison results of GC content and histone modification signals between the high interactive OCRs identified by PlantCTCIP and the control group. (H) Based on the sequence importance gradient values calculated using DeepLIFT and integrated gradients, the number of important motifs identified by PlantCTCIP in PDIs of maize, cotton and wheat. (I) The core seq logo of the 5 categories obtained by clustering the identified motifs.

We firstly obtained the distinct genome regions potentially involved in PDI in the whole genome. Specifically, the Open Chromatin Regions (OCRs) of different tissues about four crops were obtained from the existing literature (Zhu et al. 2020), and we analysed the overlap between OCRs and genic regions in the four plants (Tables S6 and S7). In maize, the length of most OCRs is within 500 bp (Figure S12). For the OCRs shorter than 1.5 kb, we obtained 1.5 kb OCR sequences by extending both flanking ends based on the reference genome. Then, we did the chromatin interaction predictions for each OCR with genes within a range of 2 Mb upstream and downstream of the OCR in the reference genome of maize, cotton and wheat. The results are shown in Figure 5A–C, respectively (Figure S13 and Table S8). Similar to the analysis method in PPIs mode, we further validated the predicted PDIs in the regions of chr1: 4.7–8.7 Mb and chr2: 1.1–5.1 Mb using Hi‐C experiments in the maize seedling stage. The results elaborate the reliability of the prediction results of PlantCTCIP (Figure 5D,E). We define the number of genes associated with each OCR in the predicted PDIs as the interaction degree of that OCR. We further selected the top 1500 OCRs with high interactivity for feature analysis. The 50 random sampling results showed that high interactive OCRs (HI‐OCR) have higher GC content, higher chromatin open state and enriching more active histone modification signals than the random OCRs (Figure 5F,G).

To explore the molecular characteristics of distal regulatory regions identified by PlantCTCIP in PDIs mode, we further filtered the predicted top 1500 OCRs with higher interaction degree. We obtained 83 OCRs using the following screening conditions (Supporting Information, Figure S14): high interactive degree, high expression of the regulated genes and located in chromosomal terminal regions. The results show that these OCRs exhibit distinct gradient distribution patterns for regulating different genes, and the regulated genes generally display high gradient values within the 250 bp downstream region of the transcription start site (TSS). It can be seen that specific distal regions may have different functional sites when regulating different genes. Based on the reported eQTL dataset (Liu et al. 2020), we identified a key region (chr6: 167 167 767–167 169 266) among the 83 OCRs mentioned above. The eQTL regulates three downstream genes: *Zm00001d038927*, *Zm00001d038928* and *Zm00001d038929* (Figure S15). The prediction results of PlantCTCIP show that there is interaction between this region and the three genes, and the 200 bp subregion within this region has a high gradient value. In addition, the 200 bp subregion has high chromatin openness in all 9 tissues of maize, further supporting its potential as an enhancer to play regulatory functions.

Based on the predicted 1500 highly interactive OCRs, we further identified critical motifs that influence PDIs. We leveraged interpretable methodologies of DeepLIFT and integrated gradients to identify 3795, 3944 and 619 key motifs in maize, cotton and wheat, respectively. The majority of these motifs were species‐specific, with 170 conserved motifs in all the three crops (Figure 5H). Five core motif categories can be obtained through the clustering analysis of the 170 conserved motifs (Figure 5I). Similarly, the results showed that the distribution characteristics of the 170 conserved motifs identified in the PDI mode in the genome were also divided into 5 categories (Figure S10 and Table S3). It can be seen that most of the motifs are enriched around 250 bp downstream of the TSS of the gene that has an interaction relationship with the distal element, consistent with prior reports (Washburn et al. 2019). We further utilised the TOMTOM tool (Bailey et al. 2015) to match these motifs with the PlantTFDB database. The results demonstrated high consistency between the key motifs identified by PlantCTCIP and the previously reported findings, thereby validating the reliability of PlantCTCIP in identifying functional regulatory elements in PDI mode.

According to the previously published articles and databases, 10 important motifs are detected to be involved in regulating chromatin interaction, gene expression and plant growth and development, etc. For instance, the motif AAATTAMAA is reported to affect the response of plants to adversity, located in the promoter of carotenoid dioxygen cleavage gene (AgCCD4), and regulates carotenoid degradation (Wang et al. 2021). In addition, the motif GAAGAAGA is a binding site of two shear enhancers, SR45 and SCL33, that are involved in protein translation folding during seed germination (Bai et al. 2021). A more important motif is CAACCAA, which is a MYB binding site that regulates the growth and development of cassava and responses to environmental stress (Xiao et al. 2022) (Table 2).

**TABLE 2:** The concrete information of the identified motifs of the top 1500 highly interactive distal regions in PDI mode.

Motifs	Seq‐logo	Functions	References
TATATATATA	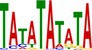	RNA polymerase binding sites determine the initiation of transcription.	Tong et al. (2022)
AAATTAMAA	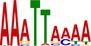	It affects the response of plants to adversity, located in the promoter of carotenoid dioxygen cleavage gene (AgCCD4), and regulates carotenoid degradation.	Wang et al. (2021)
CTAGCTAGCT	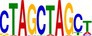	A *cis*‐regulatory element involved in the excessive response of iron in rice.	Kakei et al. (2021)
CATGCATG	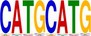	The conserved region of the seed storage protein gene promoter in leguminous plants, it binds to transcription factors and regulates the expression of storage proteins.	Bäumlein et al. (1992)
GAAGAAGA	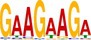	The binding sites of two shear enhancers, SR45 and SCL33, are involved in protein translation folding during seed germination.	Bai et al. (2021)
AGCTAGCT	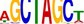	A *cis*‐regulatory elements involved in iron excess response in bitter gourd.	Cui et al. (2017)
GCWGCWGC	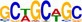	A *cis*‐regulatory element involved in iron excess response in rice.	Kakei et al. (2021)
AAGAAG	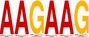	Located near the splicing site of the light regulated gene exon, it binds to transcription factors and regulates the variable splicing of introns.	Wu et al. (2014)
CAACCAA	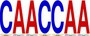	MYB binding site regulates the growth and development of cassava and its response to environmental stress.	Xiao et al. (2022)
CATGCA	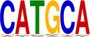	Located in the NapB (seed storage protein) promoter region, it binds to transcription factors and regulates the expression of storage proteins.	Ericson et al. (1991)

### 2.5 PlantCTCIP Can Reveal the Associations Between Transcription Factors and PDI in Multiple Plants

**FIGURE 6:**
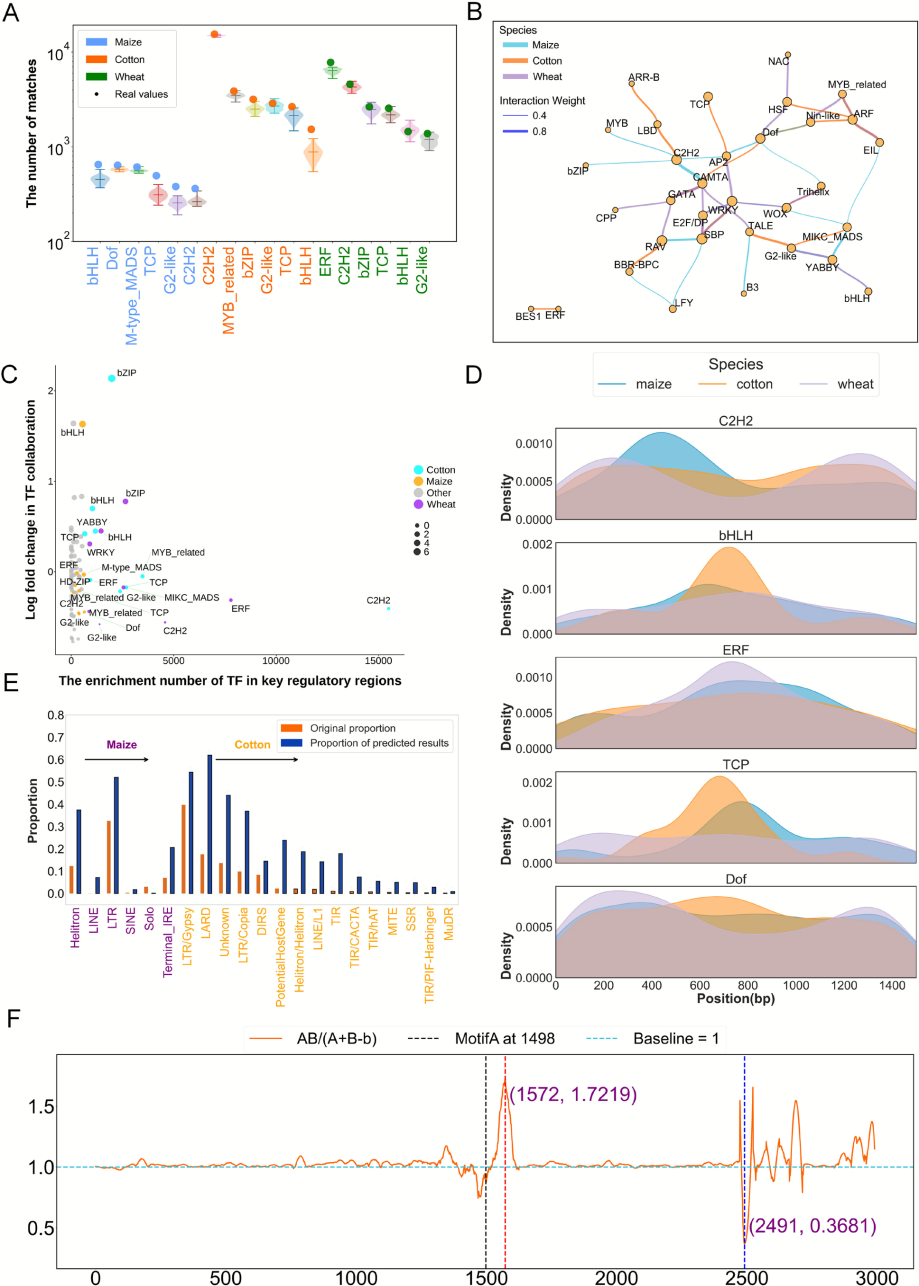
The TF enrichment difference and collaboration analysis results in the predicted multi‐species PDI sequences using PlantCTCIP. (A) The enrichment comparison results of different types of TFs (bHLH, Dof, C2H2, TCP, ERF, etc.) in regulatory sequences with high interaction and control groups. (B) Examples of TFs collaboration of maize, cotton and wheat in PDIs mode. (C) The correlation between the enrichment number of TFs in important regulatory regions and the TF‐TF collaboration of different crops. (D) The density distribution of C2H2, bHLH, ERF, TCP and Dof in important regulatory regions of three crops in PDIs mode. (E) The enrichment degree of TEs in key regulatory regions identified by PlantCTCIP is highly consistent with the distribution of the original TEs in maize and cotton. (F) The gradient value of the synergistic effect of two important motifs (ACGCC and CTGCC) on chromatin interaction in maize PDIs. In the formula of *AB/(A* + *B* − *b*), *AB* represents the gradient values of PlantCTCIP when motifs *A* and *B* collaborate. *A + B* − *b* represents the difference of the gradient value of removing motif *B*, motif *A* and the gradient of the non‐motif control (*b*).

We firstly analysed the differences of the TFs associated with high‐interactive regulatory elements in maize, cotton and wheat. We selected the distal regulatory elements with high interactivity in PDI networks and extracted their corresponding genome sequences. Subsequently, we matched the motif sequences corresponding to the reported TFs of the three plants in PlantTFDB with the top 1500 distal regulatory element sequences with high interaction degree. The distal regulatory elements with high interaction degree demonstrated significantly greater TF enrichment compared to the control group (repeat 50 times of the randomly selected 1500 distal elements) (Figure 6A). This result suggests that highly interactive distal regulatory elements may be involved in more complex gene expression regulation. Furthermore, we found that core transcription factors (CAMTA, Dof, AP2, C2H2, bHLH, bZIP and RAV) were highly conserved in the three crops. Specifically, the transcription factors of bHLH, TCP and HD‐ZIP tend to synergize with other TFs in maize. ARF and GATA primarily participated in synergistic regulation in wheat. Similarly, the transcription factors of MIKC‐MADS and B3 exhibit broader cooperative relationships with other TFs in cotton (Figure 6B). We further analysed the distribution of TFs in high‐interactive distal elements and their interacted genes in different species. The results revealed that TFs enriched in high‐interactive distal regulatory elements tend to exhibit cooperative relationships with other TFs. Notably, the transcription factors of bZIP and bHLH in cotton are not only enriched in highly interactive distal regulatory elements, but also participated in more extensive TF interactions. Although the enrichment levels of TCP and MYB‐related transcription factors were relatively low in maize, they still exhibited strong collaborative capacity. This result reflects the existence of complex regulatory mechanisms among different species or TFs (Figure 6C). We analysed the positional density distribution of 5 transcription factors (C2H2, bHLH, ERF, TCP and Dof) in maize, cotton and wheat (Figure 6D). The results demonstrated that the distribution patterns of ERF and Dof were highly similar in PDI mode of the three crops, indicating strong conservation. In contrast, bHLH and TCP were significantly enriched in cotton, while C2H2 was predominantly enriched in important regulatory regions of maize, suggesting that different species may employ distinct regulatory mechanisms of their transcriptional regulatory networks.

We further analysed the distribution of transposons within high‐interactive key regulatory regions identified by PlantCTCIP. The results indicated that key regulatory regions were predominantly enriched with the transposons of Helitron and LTR in maize, while LARD and Gypsy in cotton (Figure 6E). These findings further reflect the species differences of important regulatory elements. To further investigate the impact of key motif collaboration on chromatin interactions, we took the two important motifs identified in maize (ACGCC and CTGCC) as examples for analysis. Specifically, we evaluated the importance of these two motifs at different positions in PDI sequences by calculating gradient value changes. The results demonstrated that the PDI interaction probability of PlantCTCIP reached the maximum when the motif ACGCC was positioned at 1498 bp in PDI sequences. We further conducted positional sliding analysis on the motif CTGCC. When the motif is located at positions of 1572 and 2491 bp, PlantCTCIP obtains the largest gradient value changes, and the two positions correspond to enhancing and inhibiting PDI, respectively (Figure 6F; Figure S16; Section 4). In order to further validate the universality of the aforementioned synergistic regulation of motifs, we randomly selected three motif pairs (CACCA‐ACGCC, ACGGC‐CACCC and ACGCC‐CCTCC) and two motif pairs (CACCC‐CGCTGC and CCCGA‐ACGCC) with high weight for the same analysis in maize PPI and PDI modes (Figure S17). The results showed that the above motif pairs exhibited significant synergistic regulatory features in both modes. This may be related to the important role played by the cooperation between specific transcription factor binding sites in gene expression regulation (Figure S17). These results indicate that PlantCTCIP can not only identify potential regulatory motifs, but also localise their functional sites in specific PDI sequences and facilitate the elucidation of their regulatory roles in chromatin interactions.

### 2.6 PlantCTCIP Can Accurately Identify Target Genes That Interact With Specific Regulatory Element Sequences

**FIGURE 7:**
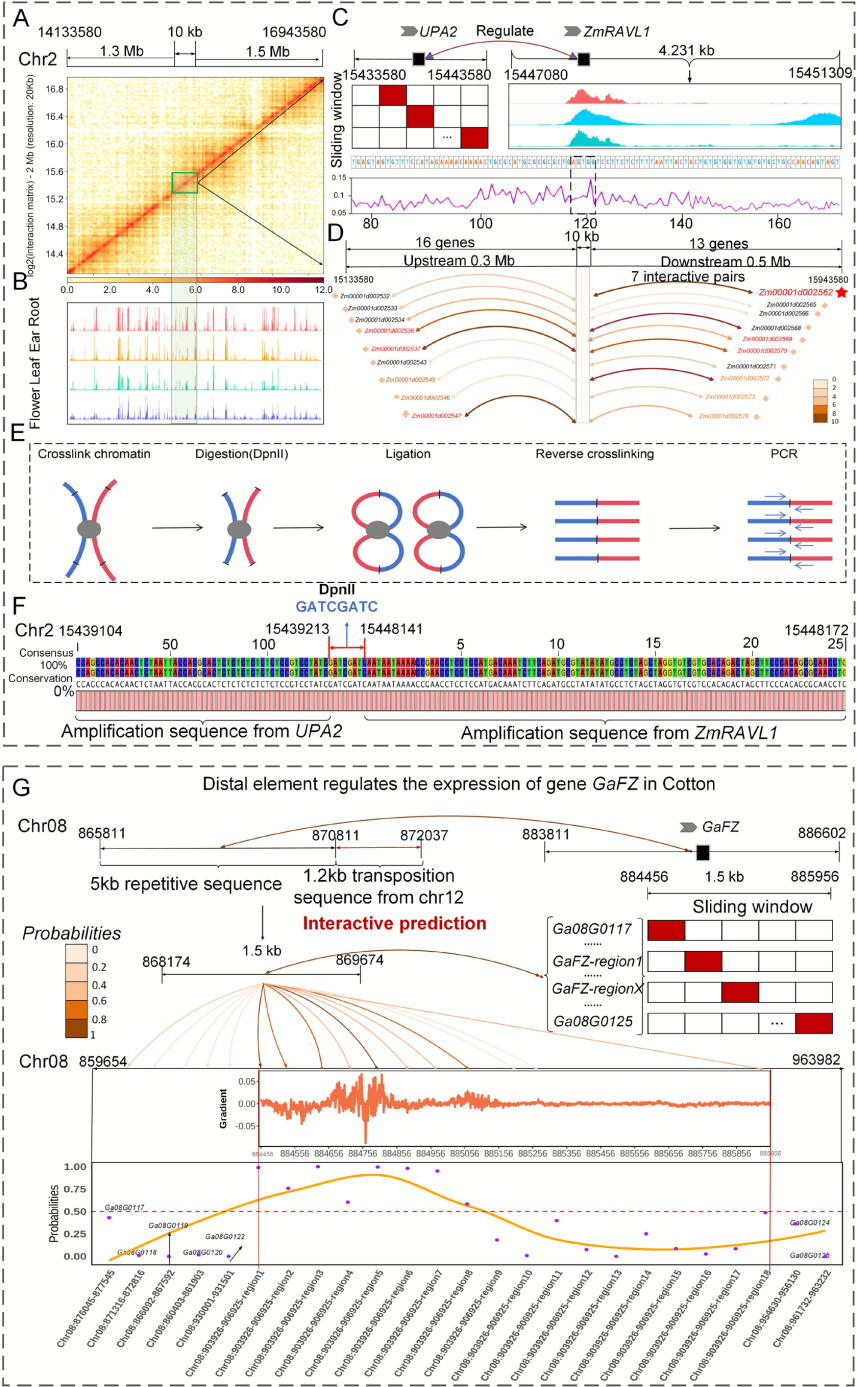
PlantCTCIP accurately identifies target genes that have chromatin interactions with specific regulatory sequences in maize and cotton. (A) The chromatin interaction pattern predicted by PlantCTCIP in the genomic region of chr2: 14 133 580–16 943 580 where maize leaf angle related gene *ZmRAVL1* is located is consistent with the Hi‐C experimental results. (B) The ATAC signals of maize multiple tissues and developmental stages in the genome region where *ZmRAVL1* is located. (C) PlantCTCIP can accurately identify the important motif AGTGC that affects the interaction of *UPA2* and *ZmRAVL1*. (D) PlantCTCIP can predict multiple genes that interact with *UPA2*, and the gene *Zm00001d002562* (*ZmRAVL1*) has the highest interaction intensity with *UPA2*. (E) Experimental process of chromatin conformation capture (3C) in leaves of maize seedlings. (F) In the 3C experiment, there exists chromatin interaction between the regulatory element (*UPA2*) and the target gene (*ZmRAVL1*) through the first‐generation sequencing. GATCGATC is the connection site after enzyme digestion. (G) PlantCTCIP can accurately identify the interaction between gene *GaFZ* and its long‐range regulatory elements in Asian cotton A2.

This section aims to further verify that PlantCTCIP can identify target genes that interact with specific regulatory element sequences. Several reported genes of maize and cotton were employed as examples to do validation and analysis. The 200 kb resolution chromatin interaction patterns and chromatin openness signals in the genomic region (chr2: 14 133 580–16 943 580) related to gene *ZmRAVL1* of maize leaf angle are depicted in Figure 7A,B. The distal regulatory element *UPA2* is known to regulate the downstream gene *ZmRAVL1* (Tian et al. 2023). In this study, we selected the *UPA2* region (chr2: 15 433 580–15 443 580, 10 kb length) located 9.5 kb upstream of *ZmRAVL1* as the distal regulatory sequence. We expanded the *UPA2* region by 0.3 Mb upstream and 0.5 Mb downstream in the reference genome, identifying 29 genes in this region (16 genes in the upstream and 13 genes in downstream regions). Subsequently, we divided the *UPA2* region into 43 sub‐regions (window size 1.5 kb, step size 0.2 kb) and extracted the genomic sequence of each sub‐region. Then we regarded the 43 sub‐region sequences and 1.5 kb sequences of the 29 candidate genes as input for the PlantCTCIP model to do interaction prediction. The results demonstrated that *Zm00001d002562* (*ZmRAVL1*) exhibited the strongest interaction strength with *UPA2*, which is consistent with the previously reported results (Figure 7D; Table S9). The DeepLIFT method was employed to calculate gradient values within the 180 bp region of *ZmRAVL1* sequence. The results revealed that the gradient value of the base sequence ‘AGTGG’ was the highest, consistent with the results reported in previous studies (Tian et al. 2019) (Figure 7C). These results provide additional evidence that PlantCTCIP can accurately identify functional sites that influence chromatin interactions. We further validated the predicted interaction of *ZmRAVL1* and *UPA2* through chromatin conformation capture (3C) experiments, confirming the prediction accuracy of PlantCTCIP (Figure 7E). The experimental results confirmed the existence of interactions between *ZmRAVL1* and *UPA2* as predicted by PlantCTCIP (Figure 7F; Figure S18). Similarly, we validated chromatin interactions associated with maize flowering stage related gene of *ZmRap2.7*. The results demonstrated that PlantCTCIP can accurately identify the interaction between *ZmRap2.7* and its regulatory element *Vgt1* (Figure S19, Table S10). To further assess the predictive capability of PlantCTCIP, we conducted validation analysis using the gene *ZmRPG*, which is involved in maize kernel dehydration. This gene encodes a 31‐amino acid polypeptide microRPG1 that controls kernel dehydration rates by regulating the expression of genes in the ethylene signalling pathway (Yu et al. 2025). We extracted genome sequences of regulatory elements *qKDR1* (chr1: 20 007 756–20 009 256) and *ZmRPG* (chr1: 20 018 612–20 021 612), then used PlantCTCIP to predict they are interacted or not. The results show that *qKDR1* exhibited the highest interaction probability (*Probability* = 0.93) with the subregion of 20 022 543–20 023 987 bp within the *ZmRPG* regions (Figure S20A–D).

Additionally, we employed the PlantCTCIP prediction model constructed from cotton A2 to validate the gene *GaFZ* and its regulatory sequence associated with cotton fibre and trichome development. Previous research has demonstrated that a 6 kb distal enhancer located at approximately position 887 151 on chromosome 8 in Asian cotton A2 can specifically activate the expression of *GaFZ*, which is a gene involved in fibre and trichome development (Wang et al. 2021). We defined a subregion chr8: 865 811–872 037 of approximately 6.3 kb upstream of *GaFZ* as the distal regulatory region and employed a sliding window approach (window = 50 bp, step = 50 bp) to divide the 6.3 kb region into multiple subsequences. Subsequently, we input these subsequences with their upstream and downstream genes (*Ga08G0117*, *Ga08G0118*, *Ga08G0119*, *Ga08G0120*, *Ga08G0122*, *Ga08G0124*, *Ga08G0125*, etc.) into the PlantCTCIP model to do interaction prediction. The results show that PlantCTCIP can accurately predict the long‐range interaction between *GaFZ* and its upstream target regions, thereby supporting the previously reported results of distal element regulating the expression of *GaFZ* (Figure 7G). In all, PlantCTCIP has demonstrated robust reliability and significant potential for identifying functional regulatory elements that interact with specific genes through chromatin interactions.

### 2.7 PlantCTCIP Provides a Real‐Time Online Website

**FIGURE 8:**
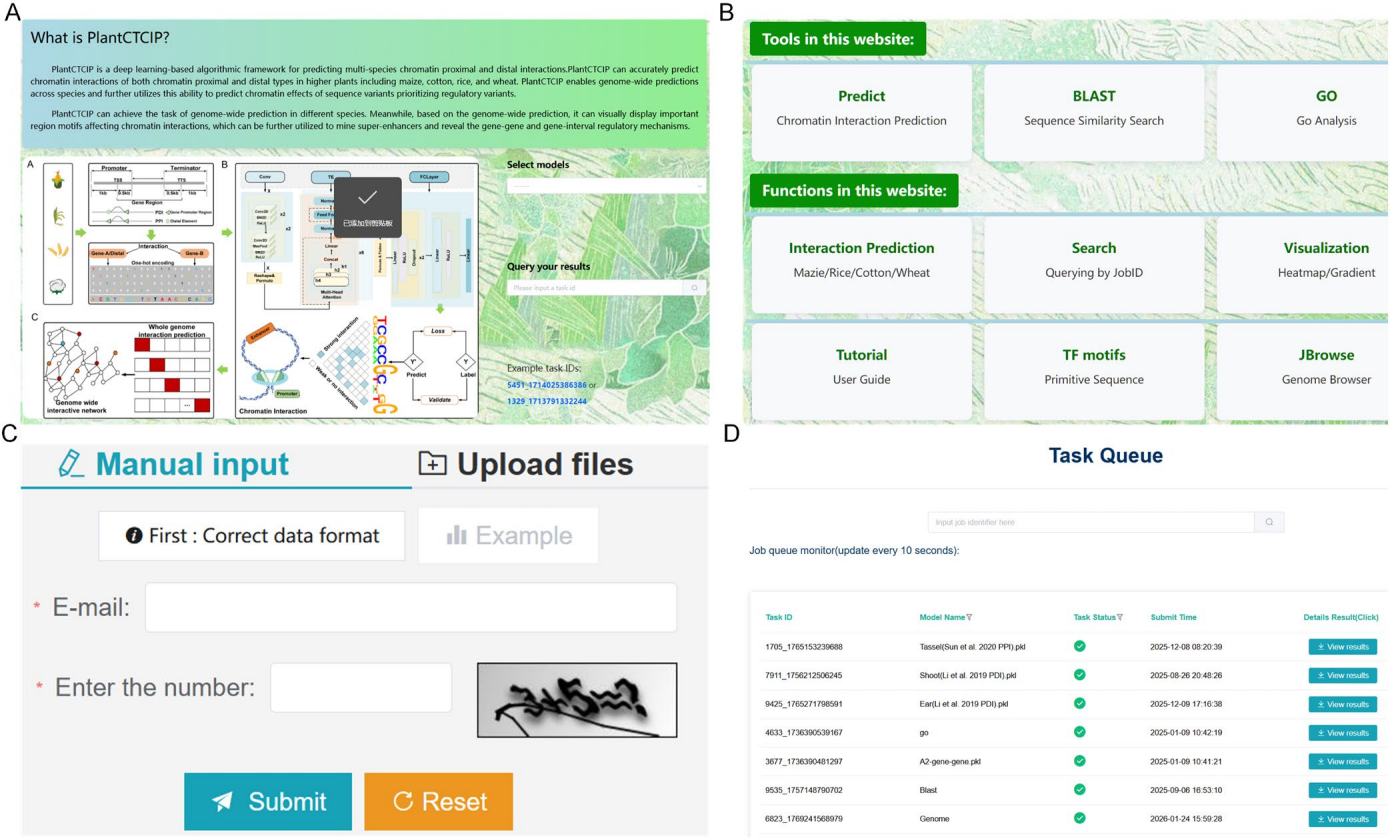
The online website of PlantCTCIP. (A) The functions of PlantCTCIP online website. (B) PlantCTCIP provides the function of high‐precision chromatin interaction prediction of four crops: maize, rice, cotton and wheat. Users can freely select the corresponding models to achieve the chromatin interaction prediction tasks. (C) PlantCTCIP implements the parallel computing algorithm. The prediction results are sent to users via email and users can view the results according to the Job_id. (D) PlantCTCIP provides a visualisation interface to display the gradient importance of the input chromatin interaction sequences.

In order to facilitate the usage of the developed PlantCTCIP model by researchers, we have developed the first real‐time online chromatin interaction prediction platform of multiple plants (http://www.plantctcip.com). The platform integrates three‐dimensional genome data of multiple tissues about four plants: maize (B73), rice (MH63, ZS97), wheat (Chinese Spring) and cotton (A2, B1, C1, D5, E1, F1, G1, K2). It can achieve accurate chromatin interaction predictions of two modes (PPIs and PDIs) in four crops. At the same time, the platform introduces an interpretable deep learning module that utilises the DeepLIFT algorithm to generate a heat map of base importance in chromatin interaction sequences. This heat map visualises the importance distribution of regulatory elements (such as *UPA2*) in chromatin interactions (Figure 8).

## 3 Discussion

### 3.1 Performance of PlantCTCIP

This study mainly carries out the following aspects of work: (1) We developed PlantCTCIP, a deep learning model for high‐precision prediction of chromatin interactions of multiple plants. PlantCTCIP firstly employs CNN to extract local sequence features and then utilises transformer architecture to capture long‐range relationships among features, thereby enhancing prediction accuracy. (2) PlantCTCIP achieved the highest accuracy in predicting chromatin interactions in maize, rice, wheat and cotton. Compared with the existing methods, PlantCTCIP demonstrated an average improvement of 14.56% in AUC values of four crops in PPIs mode. Similarly, PlantCTCIP exhibited an average improvement of 9.6% in AUC values of three crops in PDIs mode. (3) We used PlantCTCIP to construct the genome‐wide PPI and PDI chromatin interaction maps of four crops: maize, rice, wheat and cotton. We analysed sequence characteristics of highly interactive genes and distal regulatory elements, including eQTLs and chromatin accessibility signals. We explored motifs that influence PPIs and PDIs and conducted comparative analyses of motifs and their corresponding transcription factors of different crops. (4) PlantCTCIP can accurately predict target genes that have interactions with the distal regulatory elements, and we validated these prediction results using 3C experiments. In practical applications, we recommend selecting tissues that match the data used to train PlantCTCIP for predicting the related tasks. Adopting this strategy is essential to effectively enhance chromatin interaction prediction performance of the target tissue. When the data of a specific tissue is scarce, PlantCTCIP's cross tissues prediction generalisation ability can be fully utilised to verify the interaction between genes and distant elements in the target tissue. Additionally, we calculated gradient values that reflect the importance of individual bases in the PPI and PDI sequences, thereby facilitating precise localization of functional sites in regulatory elements. PlantCTCIP enables accurate identification of functional genes that regulated by specific distal elements, and contributes to the analysis of molecular design breeding. (5) To facilitate the use of PlantCTCIP model by biological researchers, we have developed a real‐time online service website (http://www.plantctcip.com).

### 3.2 Robustness and Limitations of Predicting Chromatin Interactions Across Species

In this study, we validated the generalisation ability of PlantCTCIP using the datasets of different developmental stages and tissues in maize. The experimental results indicate that PlantCTCIP has highly accurate chromatin interaction prediction results although there are significant tissue‐specific differences between the training and the predicted data. It fully demonstrates that PlantCTCIP has good robustness and generalisation ability when predicting chromatin interactions across tissues and stages (Figure S7). However, there is significant improvement room in the performance of PlantCTCIP in predicting chromatin interactions across specific tissues and stages. On one hand, the batch effect caused by different experimental procedures results in varying chromatin interaction prediction accuracy within the same tissue. Generally, the prediction accuracy of different tissue datasets from the same experiment is relatively high. For example, Shoot (Peng et al. 2019) and Shoot (Li et al. 2019) are both maize seedling tissues, but their chromatin interaction prediction accuracy is lower than that of another experimentally derived dataset: Shoot (Li et al. 2019) and Ear (Li et al. 2019). On the other hand, chromatin structure exhibits tissue specificity across different tissues. The chromatin three‐dimensional structures of Ear and Tassel (Sun et al. 2020) and Ear and Shoot (Li et al. 2019) from the same batch of experiments exhibit tissue and stage specificity. When we use data from one tissue (such as Shoot) to predict another tissue with significant differences (such as Ear), some tissue‐specific chromatin interactions will not be captured. It will lead to a decrease in the prediction accuracy of PlantCTCIP. Therefore, stronger domain adaptation strategies are needed in the future to reduce data batch effects when integrating multi‐source data. It will help improve the chromatin interaction accuracy of PlantCTCIP across tissue and stages.

### 3.3 Challenges of Predicting Chromatin Interactions Across Species

However, chromatin interaction data of different tissues and materials in plants remains limited, which directly impacts the performance of chromatin interaction prediction models. For instance, PlantCTCIP exhibits significantly different prediction accuracies for different crops. The prediction accuracy of wheat and cotton is substantially lower than that of maize and rice. Taking hexaploid wheat as an example, its reference genome size is approximately 17 Gb. It will substantially expand the non‐interacting genomic regions that constitute the negative sample space, leading to extreme class imbalance between positive and negative samples. The wheat genome is enriched in repetitive sequences, resulting in an extremely sparse distribution of true chromatin interaction signals and a markedly reduced overall signal‐to‐noise ratio. Although the training set of wheat includes a large number of genes, the coverage of informative (positive) features remains insufficient relative to the vast genomic search space. Therefore, we consider the number of high‐confidence positive samples and the intrinsic signal‐to‐noise ratio of the genome to be the key determinants of model prediction accuracy. For the complex genomes of wheat, a larger number of training datasets are required to mitigate the confounding effects of background noise. Additionally, PlantCTCIP has higher false positive or negative rates for some specific chromatin interactions.

In addition, the experimental results indicate that transfer learning can improve the prediction accuracy of PlantCTCIP to a certain extent when predicting chromatin interactions across species. However, there is still significant room for improvement in the prediction accuracy across species using PlantCTCIP, and the accuracy of cross‐species prediction is lower than that of intra‐species prediction. The accuracy of transfer learning between maize and rice is better than that between cotton and wheat (complex allohexaploid). The reason may be related to the following two aspects. The first one is the distance of the species in the evolutionary tree. Experimental results show that transfer learning achieves the best predictive performance between maize and rice, both belonging to the Poaceae family. In contrast, transfer learning has lower accuracy in predicting chromatin interactions about cotton and wheat. It indicates that there are significant differences in genome sequence features and regulatory elements that affect chromatin interaction in monocotyledonous and dicotyledonous plants. The second one is the difference of chromosomal ploidy. Wheat has a large genome and multiple homologous chromosomes, which results in generally low accuracy in predicting chromatin interactions related to wheat (allohexaploid). Constructing a large‐scale chromatin interaction model that considers both the evolutionary distance of the genome and chromosome ploidy may be one of the paths to address the aforementioned problems. The experimental results showed that PlantCTCIP identified fewer important motifs in both PPI and PDI modes of wheat compared to other species. On the one hand, it may be related that wheat is a heterozygous hexaploid species with a high proportion of repetitive sequences in its genome. Sequence similarity makes it difficult to uniquely locate Hi‐C sequencing reads. A large amount of sequence data is filtered out during the quality control stage, significantly reducing the density of effective chromatin interaction signals. On the other hand, sequence redundancy between sub‐genomes can influence the construction of background models in motif enrichment analysis. It will mask some low‐abundance or sub‐genome‐specific real motif signals, thereby reducing the sensitivity of regulatory element recognition. Therefore, it is imperative to expand sample sizes and optimise feature engineering strategies to further enhance the prediction performance of PlantCTCIP.

### 3.4 Future Perspectives

The coding regions constitute only a small fraction of plant genomes. Many non‐coding regions contain regulatory elements of promoters and enhancers, which regulate genome activity through the three‐dimensional spatial organisation of chromatin. Currently, chromatin conformation capture technologies (such as Hi‐C and ChIA‐PET) have revealed complex long‐range chromatin interaction patterns in crops. However, the researches of this area still face multiple limitations. On one hand, the mechanisms of the non‐coding regulatory elements controlling specific genes through chromatin folding remain incompletely understood. Compared to the studies in human, the three‐dimensional genome research in plants is still in its infancy. Chromatin interaction data for the model plants of maize, rice and arabidopsis remain relatively limited, which constrains comprehensive cross‐species analyses of chromatin interactions. Additionally, most current chromatin interaction prediction models are developed based on data of humans and mice, and they cannot be directly used into plants. Furthermore, the complexity of plant genomes, high proportion of repetitive sequences and frequent polyploidization events all impose greater demands on prediction models. Therefore, it is essential to develop the chromatin interaction prediction models with broad plant adaptability and high generalisation capability. Integrating multidimensional features of sequence conservation, epigenetic modifications and chromatin accessibility can enhance the prediction accuracy of chromatin interaction in plants. These measures not only facilitate the elucidation of regulatory mechanisms underlying non‐coding three‐dimensional genome, but also have the potential to advance comprehensive genome research of crops.

Simultaneously, the continuous advancement of various sequencing technologies has substantially accelerated the development of epigenomics. At present, a large amount of data related to chromatin open regions, transcription factor binding, DNA methylation and histone modifications of different plants has been accumulated. These resources provide a robust foundation for integrating and analysing multi‐omics data. The continuous optimization of artificial intelligence algorithms, particularly deep learning, also offers powerful technical support for understanding the dynamic regulation of chromatin interaction networks. Looking ahead, the accuracy of predicting three‐dimensional genome structures across tissues and species is expected to be significantly improved. Researchers will also conduct more systematic and comprehensive analyses of chromatin interactions mediated by non‐coding regulatory elements (such as enhancers, insulators, etc.). In the future, we will mainly focus on the following research aspects: (i) Expanding chromatin interaction datasets of multiple tissues and plants to enhance the generalisation capacity of prediction models. (ii) Integrating epigenetic data, including chromatin accessible regions, DNA methylation and histone modifications, to develop more comprehensive and accurate chromatin interaction prediction models. (iii) Combining AI technologies to investigate dynamic changes of chromatin interactions at the single‐cell level, thereby revealing cellular heterogeneity and regulatory mechanisms across different developmental stages. (iv) Further improving the functions of the online service platform to achieve more user‐friendly interactive visualisation of sequence gradient results and more multi‐omics data analysis, and promoting the practical application of PlantCTCIP in biological analysis.

## 4 Materials and Methods

### 4.1 Data Analysis

#### 4.1.1 Overview of the Datasets

Taking maize B73 material as the research object, this study integrated maize chromatin interaction data obtained using ChIA‐PET and ChIP‐Seq technologies (Peng et al. 2019; Li et al. 2019; Sun et al. 2020). Additionally, this study used the multi‐omics data of TFs, TFBS, gene expression and eQTLs to conduct experiments. The maize B73 reference genome and annotation files were obtained from the NCBI database (Jiao et al. 2017). The TF and TFBS data were retrieved from the PlantTFDB database (Jin et al. 2016), and eQTLs data were obtained from Liu et al. (2020). The Rice dataset involves the chromatin interaction data of two published accessions of MH63 and ZS97 (Zhao et al. 2019; Zhang et al. 2016). The PPI and PDI data of wheat (Chinese Spring) were from Yuan et al. (2022). The cotton‐related data includes chromatin interactions and transposon data of eight accessions: A2, B1, C1, D5, E1, F1, G1 and K2 (Pei et al. 2022). The detailed information of all the datasets is shown in Table S1.

#### 4.1.2 PDI Data Processing

The PDI datasets of shoot tissue comprising 11 207 PDIs (Li et al. 2019). The intergenic distal sequences of different tissues ranged in length of 1 to 2 kb, and a uniform length of 1.5 kb was applied to all distal sequences. The promoter proximal region was also 1.5 kb, including 1 kb upstream and 0.5 kb downstream of the gene transcription start site (TSS). For the distal sequences shorter than 1.5 kb, *N* bases were added to both ends to reach 1.5 kb. When the length of the distal sequences exceeded 1.5 kb, a central 750 bp region was extracted in both directions to generate 1.5 kb sequence.

#### 4.1.3 OCRs Data Processing

For the PDI dataset, we firstly analysed the length distribution of the reported OCR data across different maize tissues (Zhu et al. 2020). The results revealed significant differences in OCR lengths among different tissues, with most OCRs being < 500 bp in length (Figure S12). Furthermore, we calculated the distance (denoted as *D*) between each OCR and its nearest gene and plotted the density distribution of *D* values. The results indicated that there were significant differences in the distance between OCRs and the nearest gene across different tissues, demonstrating that the spatial distribution of OCRs in the genome is tissue‐specific (Tables S6 and S7).

#### 4.1.4 Construction of a Genome‐Wide PDI Chromatin Interaction Dataset

Firstly, we selected OCRs located larger than 2 kb away from specific genes as distal regulatory elements (denoted as distal). Subsequently, each distal region was expanded by 1 Mb on both sides, and all genes within the expanded regions (totaling 2 Mb+) were selected as the candidate genes that are regulated by the OCR. The sequence of each distal region and candidate genes are input into PlantCTCIP to do prediction. The process of predicting genome‐wide PDIs include the following steps. Based on the distance between OCRs and genes, the OCRs with distances > 2 kb were firstly selected as the distal elements, and their starting and ending positions were denoted as *S*_*0*_ and *E*_*0*_. Next, the region was expanded by 1 Mb to the left and right, obtaining new starting and ending positions *S*_*1*_ = *S*_*0*_ − 1 000 000 and *E*_*1*_ = *E*_*0*_ + 1 000 000. All genes in the region [*S*_*1*_, *E*_*1*_] were denoted as *M*. Subsequently, the current distal region was combined with *M* genes to obtain *M* chromatin interaction sample pairs, denoted as [distal, *Gene*_*m*_], *m*∈*M*. The sequences of each sample pair were encoded and input into PlantCTCIP for chromatin interaction prediction, obtaining the results [distal, *Gene*_*i*_, label]. The label indicates whether there is interaction or not (0 represents no interaction, 1 represents interaction).

#### 4.1.5 Prediction of PPIs and PDI in Specific Genome Regions

According to the reported methods (Fudenberg et al. 2020), this study achieved chromatin interaction prediction in specific chromosomal regions in both PPI and PDI modes of four plants. Taking maize as an example, the specific process of PPI mode is shown as follows: (i) The specific genomic region was divided into multiple Bins according to a window size of 1.2 Mb and a step size of 1.2 Mb. (ii) Each bin was divided into 400 subsequences with a window and step size of 3000 bp. (iii) Combine these 400 subsequences pairwise to obtain a matrix *M* containing 160 000 sample pairs. The chromatin sequences of each sample pair were input into the PlantCTCIP model for PPI prediction. The predicted results were in the format of [*Gene_A*, *Gene_B*, label], where label = 1 indicates there exists interaction of the two genes, and label = 0 indicates no interaction. Then, it generates the chromatin interaction heat map of the specific chromosomal region based on these results. For the PDI mode, the specific chromosomal regions were divided into several bins based on a window size of 0.6 Mb and a step size of 0.6 Mb. Each bin was divided into 400 subsequences with a window size of 1500 bp and a step size of 0.6 Mb. Similarly, these 400 sequences were combined pairwise to obtain a matrix *M* containing 160 000 sample pairs, which are encoded and input into the PlantCTCIP model for PDI prediction. Similarly, a chromatin interaction heat map of the specific region can be constructed based on the predicted results. Figures S21 and S22 show two examples of the prediction results in two representative bins of maize PDI mode using PlantCTCIP. The published multi‐omics data elaborates that PlantCTCIP is capable of identifying interacting sequence intervals in chromosomes.

### 4.2 Methods

#### 4.2.1 Framework of PlantCTCIP

The PlantCTCIP model uses one‐hot encoding, and it includes two modules: CNN and Transformer. The CNN module includes three parts, in which there are two convolutional layers. Each convolutional layer connects to a maximum pooling layer to achieve feature dimensionality reduction and feature re‐extraction. Transformer is used to capture feature information about the proximal and distal chromatin sequences and then mine important motif features that affect chromatin interactions. To reduce overfitting, the mechanisms of batch normalisation and dropout are used. In the last layer of PlantCTCIP, the sigmoid or linear activation functions are used to do prediction (Figure 1B).

Taking the PDI model as an example, this study extracted the upstream 1 kb and downstream 0.5 kb sequences from the gene TSS as the sequence of each gene. Due to the different lengths of distal element sequences (*D*_*seq*_) that interact with target genes, we adopted a truncation and padding approach to process distal regulatory sequences. For the distal regulatory sequences (*D*_*seq*_) with length < 1.5 kb, we obtained the sequences with equal length of *P*_*seq*_ by padding at both ends. For the distal element sequences (*D*_*seq*_) with length > 1.5 kb, the sequences were truncated by expanding symmetrically from the current sequence center until reaching 1.5 kb.

(1) Using the commonly used One‐hot encoding method to process *P*_*seq*_ and *D*_*seq*_, A = [1, 0, 0, 0]^T^, T = [0, 1, 0, 0]^T^, G = [0, 0, 1, 0]^T^, C = [0, 0, 0, 1]^T^, N = [0, 0, 0, 0]^T^. The encoded sequences of gene and distal region are denoted as $P_{\mathrm{seq}}\in R^{4*1500}$, $D_{\mathrm{seq}}\in R^{4*1500}$. Then we concatenated *P*_*seq*_ and *D*_*seq*_ and input them into the model, *DP* = *Concat* (*D*_*seq*_, *P*_*seq*_), $\mathrm{DP}\in R^{4*3000}$ (Figure 1A).

#### 4.2.2 CNN Layer

In PlantCTCIP, the convolution operation is firstly used to perform dimensionality reduction and extract important features. For an encoded matrix $\mathrm{DP}\in R^{4*3000}$ and a filter $W\in R^{U*V}$, U<<4,V<<3000, the convolution operation is shown in Equation (1).

(1) $$S_{\mathrm{ij}}=\sum_{u=1}^{U}\sum_{v=1}^{V}W_{\mathrm{uv}}{\mathrm{DP}}_{i-u+1,j-v+1}$$

Through the above operation, the sequence *DP* is transformed into a sequence $S\in R^{4*d}$ with lower dimensions, *S* = (*s*_*1*_, *s*_*2*_, …, *s*_*d*_), $S_{i}\in R^{4}$. *d* represents the dimension of the extracted sequence features, as shown in Figure 1B.

#### 4.2.3 Transformer Layer

In order to capture long‐range dependencies between sites in the sequence, we further utilised Transformer to capture distant features in the sequence. Transformer mainly uses multi‐head attention mechanism, consisting of multiple self‐attention modules. For *s*_*i*_ in a self‐attention module, it generates three vectors: query vector, key vector and value vector. The above three vectors are obtained by multiplying *s*_*i*_ with three weight matrices (query matrix *W* ^*Q*^, key matrix *W* ^*K*^ and target weight matrix *W* ^*V*^) $S_{i}^{q}=W^{Q}*S,\;S_{i}^{k}=W^{K}*S,\;S_{i}^{v}=W^{V}*S$. Then, it does product operation between the query vector $S_{i}^{q}$ of *S*_*i*_ and the key vectors $S_{j}^{k}$ of all elements in *S*. And it obtains the correlations of all elements $\alpha_{\mathrm{ij}}$ to the current element. All $\alpha_{\mathrm{ij}}$ are normalised using Equation (2).

(2) $$\alpha_{\mathrm{ij}}^{\prime }\;=\;\frac{\exp\left(\alpha_{\mathrm{ij}}\right)}{\sum_{j}\exp\left(\alpha_{\mathrm{ij}}\right)}$$

It multiplies $\alpha_{\mathrm{ij}}^{'}$ and $S_{j}^{v}$, and sums them up to obtain the output *S*_*i*_^*′*^ of self‐attention on *S*_*i*_, as shown in Equation (3), where $j\in \left[1,n\right]$. It performs the above operations on all elements in sequence *S*, obtains *S* ^*′*^ = (*s*_*1*_^*'*^, *s*_*2*_^*'*^, *s*_*3*_^*'*^, …, *s*_*n*_^*'*^). The above process can be calculated using the matrix to improve the efficiency, as shown in Equation (4).

(3) $${S_{i}}^{'}=\sum_{j}{a_{\mathrm{ij}}}^{'*}S_{j}^{v}$$

(4) $$\text{Attention}\left(Q,K,V\right)=\text{softmax}\left(\frac{QK^{T}}{\sqrt{d_{k}}}\right)V$$

Among them, *Q*, *K* and *V* represent the query matrix, key matrix and value matrix of sequence *S*, respectively. Each row in the matrix represents the query vector, key vector and value vector of each element, and *d* ^*k*^ represents the vector length. The multi‐head attention mechanism concatenates the outputs of multiple self‐attention events and then obtains the output through the linear transformation. The calculation method of the above process is shown in Equation (5), and Concat represents the concatenation function.

(5) $$\text{MultiHead}\left(Q,K,V\right)\;=\;\text{Concat}\left({\text{head}}_{1},\;{\text{head}}_{2},\;\ldots \ldots ,\;{\text{head}}_{h}\right)W^{O}$$

(6) $${\text{head}}_{i}=\;\text{Attention}\left(QW_{i}^{Q},\;KW_{i}^{K},\;{\mathrm{VW}}_{i}^{V}\right)$$

The global information *S* ^*all*^ of sequence *S* can be obtained through the above calculation process, and then flatten *S* ^*all*^ into one‐dimensional vector. *X* = *Flatten* (*S* ^*all*^), and $X\;\in R^{4*d}$. Finally, *X* is input into the fully connected layer to determine whether there is interaction between the gene proximal region sequence *P*_*seq*_ and the distal element sequence *D*_*seq*_ or not. The above calculation process is shown in Figure 1B. The parameter settings and implementation process of PlantCTCIP are shown in Figure 1, Table S11 and Algorithm S1. The batch size, learning rate, dropout, number of convolutional filters, the size of convolutional kernels are the hyperparameters of PlantCTCIP. Experimental results show that the batch size has large impact on the prediction results (Figure S23). The model's prediction accuracy also improves as the number and size of convolutional kernels increase. Through comprehensively considering the running time and memory space, the number and size of convolution kernels are set to 64 and 8 in our experiments (Figure S24). The detailed hyperparameters in the experiments are shown in the following: Learning rate = 0.0001, Dropout = 0.3, Batch size = 64. In terms of architecture, the encoder adopts a stage‐wise depth configuration of {4, 4, 8, 6}. The channel dimensions of the feature maps are sequentially set to 1, 64, 128, 256, 128, 64 and 4. Additionally, the convolutional layers are configured with a kernel size of 8 and a base filter number of 64.

#### 4.2.4 Four Comparison Models

(1) DeepTACT predicts enhancer‐promoter interactions using CNN, BiLSTM and attention mechanisms based on DNA sequences (Li et al. 2019); (2) ChINN identifies genome‐wide open chromatin interactions using CNN based on Hi‐C and ChIA‐PET data (Cao et al. 2021); (3) SEPT employs CNN, LSTM and transfer learning to predict enhancer‐promoter interactions in different human cell lines (Jing et al. 2020); and (4) SPEID predicts enhancer‐promoter interactions using CNN and LSTM based on enhancer and promoter sequences (Singh et al. 2019).

#### 4.2.5 PlantCTCIP Mines Important Sequence Regions and Motifs

DeepLIFT was used to calculate the gradient value of each base in the input sequence. Then, we use the continuous gradient method to mine important motifs.

For *Gene_A* and *Gene_B* involved in PPIs, the 3 kb sequences of the two genes are denoted as *Seq_A* and *Seq_B*, respectively.

(1) Concatenate *Seq_A* and *Seq_B* into a 6 kb sequence, which will be encoded and input into the PlantCTCIP model to do prediction.

(2) The DeepLIFT is employed to calculate the sequence gradient *M* for *Gene_A* and *Gene_B*. *M* is a 4*6000 matrix.

(3) There are 4 rows and 6000 columns in *M*, and 4 rows represent the four bases A/T/C/G. Then it scans the matrix *M*. When the gradient value of a sequence of continuous length *N* is greater than the threshold *r*, then the sequence of length *N* is regarded as an important motif. When we set *r* to 0.2 and set *N* to 5, an example of gradient matrix is shown as follows:

$$\left[\begin{array}{cccccc} A & 0.02 & 0 & 0.034 & 0 & 0.2\\ C & 0.23 & 0 & 0 & 0 & 0.48\\ G & 0 & 0.28 & 0.1 & 0.59 & 0.07\\ T & 0.16 & 0 & 0.5 & 0.66 & 0 \end{array}\right]$$

According to the calculation method of the continuous gradient mentioned above, the motif of the above gradient matrix is CGTTC. It is noted that we can mine motifs with different lengths when setting different thresholds *r*.

#### 4.2.6 Motif Enrichment Analysis in the Open Chromatin Region

To mine the features of the important motifs identified by PlantCTCIP in four crops (maize, cotton, rice and wheat), the true physical location of each motif in the chromosome is firstly identified. Then, the physical locations of the identified motifs are matched to the open chromatin regions in the NAM population. The above operation is repeated 100 times using the following two controls: (1) Removing motif sequences from the PPIs and PDIs sequences and extracting sequences with equal length from the remaining PPIs and PDIs sequences. (2) Removing PPIs and PDIs sequences from the whole genome and extracting sequences with equal length from the remaining genome sequences.

#### 4.2.7 Exploring the Impact of Motif Collaboration on Chromatin Interaction Using PlantCTCIP

Based on the identified important motifs, this study further investigated the impact of motif collaboration on chromatin interactions (de Almeida et al. 2022). Firstly, the motifs identified by PlantCTCIP were matched with the databases (such as PlantTFDB, JASPAR, etc.) to obtain the matching motifs. Subsequently, these motifs were combined pairwise to construct motif‐motif matching pairs. Supposing two motifs *M*_*1*_ and *M*_*2*_, it obtains the position of *M*_*1*_ in the original interaction pair (*P*_*1*_). For example, *M*_*1*_ is located in *Gene_A*. Then, *M*_*2*_ was placed on *Gene_B* that interacts with *Gene_A*, and a sliding window method (window = step = length (*M*_*2*_)) was used to move *M*_*2*_'s position on *Gene_B* gradually. The new position of *M*_*2*_ on *Gene_B* was denoted as *P*_*i*_ for each movement, where *i* represents the number of steps. Simultaneously, the corresponding sequences of *Gene_A* and *Gene_B* were input into the PlantCTCIP model to calculate their gradient values. Finally, the position *P*_*m*_ with the largest gradient change value can be obtained. If the gradient value of the model changed significantly at this position *P*_*m*_, *M*_*2*_ was considered to have a cooperative relationship with *M*_*1*_ at *P*_*m*_. Then, *M*_*1*_‐*M*_*2*_ at the corresponding position has a significant impact on chromatin interactions (Figure 6F; Figures S16B and S17).

#### 4.2.8 Saturation Mutations in Important Regulatory Sequence Regions Based on PlantCTCIP

To evaluate the impact of key motifs on chromatin interactions, we conducted saturation mutation analysis on important regulatory sequences. Firstly, DeepLIFT and continuous gradient methods were used to identify key motifs from 83 distal regulatory sequences. Regarding the target motif as the center, it extends *N* bases (*N* = 50, 100 and 200) to the left and right sides to construct extension regions. The region was then simulated for mutation by randomly replacing the four bases with A/T/C/G. The mutated sequences are input into the PlantCTCIP model to do interaction prediction. Finally, it calculates the interaction degree of each mutated distal region and compares it with the interaction degree before mutation to obtain the impact of specific motifs on chromatin interactions. The above methods can effectively reveal the potential of key motifs for regulating distal genes, further verifying the practicality of PlantCTCIP in analysing the functions of regulatory elements.

#### 4.2.9 High Throughput Hi‐C Sequencing

To verify the accuracy of PlantCTCIP in predicting genome‐wide chromatin interactions across different species, this study conducted high‐throughput Hi‐C experiments using maize B73 seedlings material. The experimental workflow is as follows: (i) Sample fixation and detection. Maize seedling leaf tissue was crosslinked with formaldehyde to preserve the 3D architecture of chromatin. After cell lysis, cross‐linked chromatin was obtained. (ii) Chromatin enzyme cleavage. The restriction enzyme DpnII was used to cleave cross‐linked chromosomes. DpnII is a restriction endonuclease that recognises 4‐bp sites and produces uniform sticky ends after cleavage, facilitating subsequent fragment ligation. (iii) Biotin labelling and fragment ligation. First, end repair was performed on DNA fragments after enzyme digestion, and then the repaired blunt ends were labelled with biotin. The labelled fragments were ligated by T4 DNA ligase based on their spatial adjacency to form chimeric DNA reflecting chromatin three‐dimensional interactions. After ligation was completed, high‐quality Hi‐C samples were obtained by digesting with proteinase K and extracting with phenol‐chloroform to remove cross‐linking and purify DNA. (iv) Library preparation. DNA fragments were sonicated to 300–500 bp. Then, end repair was performed on the fragments and a single ‘A’ nucleotide was added to the 3′ ends (A‐tailing) (dA tailing) to connect the sequencing adapters to form Hi‐C library fragments with adapters. PCR amplification of library fragments to obtain high‐quality library products. (v) High‐throughput sequencing. To obtain a high‐resolution maize whole‐genome chromatin interaction map, a qualified Hi‐C library was subjected to 150‐bp paired‐end sequencing (PE150) using the Illumina NovaSeq 6000 platform.

#### 4.2.10 Hi‐C Data Analysis

(i) Data preprocessing: Fastp software was used to perform quality control on the raw sequencing data, removing low‐quality read segments and adapter sequences (Chen et al. 2018). (ii) Data alignment and interaction matrix generation: The filtered high‐quality reads were aligned with the maize reference genome (B73‐v4) using HiC‐Pro software (version 3.1.0) (Servant et al. 2015). After alignment, duplicated, self‐ligated and low‐quality fragments were removed, and a chromatin interaction matrix was generated with a resolution set to 40 kb. (iii) Chromatin interaction heat map generation: The interaction matrix data were visualised using the HiCPlotter tool to generate a heat map of chromatin 3D interactions, revealing the spatial tissue structure of chromatin (Akdemir and Chin 2015). (iv) Data format conversion and loop information extraction: Juicer software (version 1.9.9) was used to convert the .allValidPairs file generated by HiC‐Pro into HIC file format, and loop information of chromatin interactions was further extracted (Durand et al. 2016). (v) High‐frequency interaction segment verification: For the extracted high‐frequency interaction fragments, their corresponding sequencing reads (R1 and R2) were located and 30 bp segments were extended upstream and downstream from the enzyme cleavage sites, respectively. The extended segments were then compared with the high‐frequency interaction segments. If two 30 bp segments corresponding to the same sequence ID were aligned with two high‐frequency interaction segments, they were considered as reliably detectable regions.

## Author Contributions

Conceptualization: J.L., Y.P. and J.Y.; methodology: Z.W., Z.G., Z.N., J.Q. and R.Z.; writing – original draft: Z.W., S.Z. and Z.G.; writing – review and editing: Z.W., S.Y., Z.G., Y.P. and J.L.; funding acquisition: J.L.

## Funding

This work has been supported by the National Natural Science Foundation of China (32572406, 32400545), the Major Program (JD) of Hubei Province (2025BEA003), the National Key Research and Development Program of China (2022YFD1201504), Guizhou Provincial Basic Research Program (Natural Science) (MS[2025]096), Hubei Provincial Natural Science Foundation (2024AFB416, 2023AFB832), Major Project of Hubei Hongshan Laboratory (2022HSZD031), the Henan Province key research and development project (No. 231111110100).

## Conflicts of Interest

The authors declare no conflicts of interest.

## Supporting information


**Data S1:** pbi70586‐sup‐0001‐Data S1.Zip.

## Data Availability

Methods, including statements of data availability and any associated accession codes and references, are available at https://github.com/Zy‐Wang‐18/PlantCTCIP/tree/master. The raw sequence data (Hi‐C) are available at NCBI under accession number PRJNA1392916.
